# Steering Stem Cell Fate within 3D Living Composite Tissues Using Stimuli‐Responsive Cell‐Adhesive Micromaterials

**DOI:** 10.1002/advs.202205487

**Published:** 2023-01-04

**Authors:** Tom Kamperman, Niels G. A. Willemen, Cindy Kelder, Michelle Koerselman, Malin Becker, Luanda Lins, Castro Johnbosco, Marcel Karperien, Jeroen Leijten

**Affiliations:** ^1^ Department of Developmental BioEngineering Faculty of Science and Technology Technical Medical Centre University of Twente Drienerlolaan 5 Enschede 7522NB The Netherlands

**Keywords:** 3D cell culture, cell–matrix interactions, microgels, smart materials, tissue engineering

## Abstract

Engineered living microtissues such as cellular spheroids and organoids have enormous potential for the study and regeneration of tissues and organs. Microtissues are typically engineered via self‐assembly of adherent cells into cellular spheroids, which are characterized by little to no cell–material interactions. Consequently, 3D microtissue models currently lack structural biomechanical and biochemical control over their internal microenvironment resulting in suboptimal functional performance such as limited stem cell differentiation potential. Here, this work report on stimuli‐responsive cell‐adhesive micromaterials (SCMs) that can self‐assemble with cells into 3D living composite microtissues through integrin binding, even under serum‐free conditions. It is demonstrated that SCMs homogeneously distribute within engineered microtissues and act as biomechanically and biochemically tunable designer materials that can alter the composite tissue microenvironment on demand. Specifically, cell behavior is controlled based on the size, stiffness, number ratio, and biofunctionalization of SCMs in a temporal manner via orthogonal secondary crosslinking strategies. Photo‐based mechanical tuning of SCMs reveals early onset stiffness‐controlled lineage commitment of differentiating stem cell spheroids. In contrast to conventional encapsulation of stem cell spheroids within bulk hydrogel, incorporating cell‐sized SCMs within stem cell spheroids uniquely provides biomechanical cues throughout the composite microtissues’ volume, which is demonstrated to be essential for osteogenic differentiation.

## Graphical abstract

Cellular spheroids are used to mimic native tissues but only offer poor control over their internal microenvironment. Here, stimuli‐responsive cell‐adhesive micromaterials (SCMs) that interact and self‐assemble with cells into 3D living composite tissues are developed. The behavior of stem cell spheroids, including their lineage commitment, is controlled from within by tuning cell–material interactions via the amount, size, and temporal stiffness of SCMs.

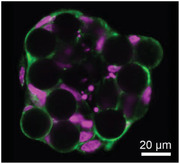

## 1 Introduction

3D cell cultures have the potential to closely emulate the architecture and function of our native tissues.^[^ ^1^ ^]^ Material‐free microtissues in the form of cellular spheroids or organoids are considered the most biomimetic 3D culture models with myriad applications including disease modeling,^[^ ^2^ ^]^ drug screening,^[^ ^3^ ^]^ and modular tissue engineering.^[^ ^4^ ^]^ 3D microtissues are typically formed via self‐assembly through cell–cell interactions within a non‐cell‐adhesive environment, such as a hanging drop, nonadhesive microwell, or spinner flask.^[^ ^5^ ^]^ Cell–cell interactions are mainly mediated by adhesion proteins called cadherins, which are of instrumental importance for tissue development, wound healing, and homeostasis.^[^ ^6^ ^]^ Cells also bind, sense, and respond to their surrounding matrix and materials via integrins.^[^ ^7^ ^]^ However, as conventional cellular microtissues are initially composed of just cells, controlling their behavior, function, and fate is challenging as they offer little control over their internal microenvironment. Specifically, cellular microtissues lack the designer cell–matrix interactions that control various important cell functions (e.g., via material elasticity or stress relaxation), which can be offered by biomaterials such as cell‐adhesive (e.g., RGD‐containing) hydrogels.^[^ ^8^ ^]^ While such biomaterials have successfully been used for 2D cell culture or encapsulation of individual cells,^[8a, 9]^ biomaterial strategies to actively control the microenvironment and guide the function of 3D cellular spheroids or organoids from within have remained largely unexplored.

Integrating cell‐adhesive micromaterials in 3D microtissues represents an elegant and effective method to homogeneously present tunable cell–biomaterial interactions within living constructs. Although cells have been combined with micromaterials based on SU‐8,^[^ ^10^ ^]^ silica,^[^ ^11^ ^]^ poly(lactic‐*co*‐glycolic) acid,^[^ ^12^ ^]^ agarose,^[^ ^12^ ^]^ poly(caprolactone),^[^ ^13^ ^]^ collagen,^[^ ^14^ ^]^ and gelatin,^[^ ^12^, ^15^ ^]^, no strategy has been designed to actively steer cell behavior via stimuli‐responsive or “smart” micromaterials. This has prevented the development of on‐demand tunable living composite tissues, which has hindered these models to provide insight into the tissue dynamicity that defines natural tissue behavior, especially during development, regeneration, and pathological onset.^[^ ^16^ ^]^ Although in situ tunable and instructive biomaterials have been explored for the biofabrication of 2D and 3D bulk materials,^[^ ^17^ ^]^ smart cell‐adhesive micromaterials have remained wanted yet elusive.^[^ ^18^ ^]^ Creating cell‐adhesive microparticles from in situ tunable biomaterials is therefore expected to expand the 3D cell culture toolbox by providing microbuilding blocks to emulate the adaptivity of native tissues within man‐made tissue constructs using a facile, modular, and scalable approach.

In this study, we established the concept of stimuli‐responsive cell‐adhesive micromaterials (SCMs) that autonomously assemble with living matter (e.g., mammalian cells) to instruct the resulting engineered tissues in a scalable, homogeneous, and on‐demand tunable manner. Specifically, the SCMs are cell‐sized integrin‐binding hydrogel microparticles (i.e., microgels) that could self‐assemble with cells into 3D living composite tissues and present biochemically and biophysically instructive cues on demand to control cellular behavior in situ. The microgels were created by microfluidic emulsification and subsequent enzymatic crosslinking of an injectable dextran‐based hydrogel functionalized with tyramine and biotin moieties (Dex‐TAB). Based on a previously reported photo‐crosslinking strategy of tyramine‐functionalized hydrogels,^[^ ^19^ ^]^ we here exploited visible‐light‐induced secondary crosslinking of tyramines using a ruthenium complex and sodium persulfate (Ru/SPS) as initiators to tune the mechanical properties of Dex‐TAB microgels inside composite microtissues. Furthermore, Dex‐TAB microgels could be chemically modified in situ in a spatiotemporal manner via a competitive supramolecular complexation strategy using avidin and biotin analogs that we have recently developed.^[^ ^20^ ^]^ Endowing Dex‐TAB microgels with cell‐adhesive moieties effectively yielded SCMs that readily formed living composite microtissues in the absence of serum. Consequently, SCMs presented temporally controlled biochemical and biophysical cues in a homogeneous manner to cellular spheroids, which was achieved using secondary orthogonal crosslinking strategies. Surprisingly and in stark contrast to material‐free microtissues and cell spheroids embedded within hydrogels,^[^ ^21^ ^]^ SCMs potently steered differentiating stem cells spheroids toward the osteogenic lineage in a material stiffness‐dependent manner. Finally, we demonstrated that SCM stiffness, size, and SCM‐to‐cell number ratio can be used to tune the osteogenesis of living composites.

## 2 Results and Discussion

### 2.1 Microfluidic Generation of Chemically and Mechanically Tunable Microgels

**Figure 1:**
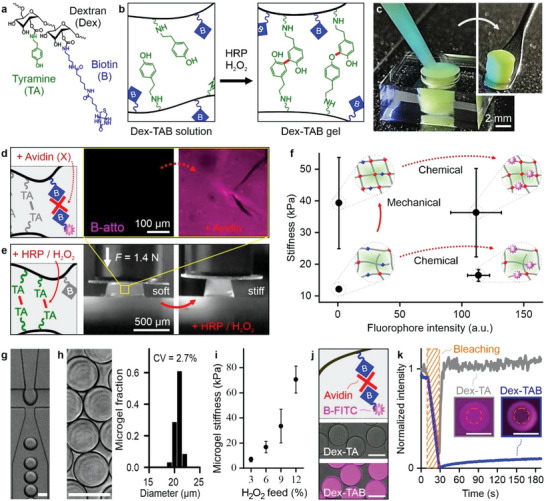
Engineering mechanically and chemically tunable microgels. a) Structural formula of dextran‐tyramine‐biotin (Dex‐TAB). b) Crosslinking of tyramine (indicated with “TA”) moieties using horseradish peroxidase (HRP) and H_2_O_2_ enabled c) injection molding of Dex‐TAB. Possible crosslinks are indicated with red bonds. d) Dex‐TAB could be chemically modified with biotinylated (indicated with “B”) moieties using an avidin‐type molecule (e.g., neutravidin, indicated with “X”) as a supramolecular crosslinker. e) Crosslinking Dex‐TAB using a substoichiometrical amount of H_2_O_2_ resulted in relatively soft hydrogels that were more mechanically compliant than the same hydrogels that underwent an enzymatic postcure as revealed by applying a compression force of 1.4 N to the hydrogel construct before and after postcuring. f) The orthogonal nature of enzymatic postcuring and biotin/avidin supramolecular complexation strategies resulted in virtually independent in situ tuning of Dex‐TAB's mechanical and chemical properties. Specifically, tyramine crosslinking did not substantially affect subsequent supramolecular functionalization of Dex‐TAB with neutravidin and biotinylated fluorophores and vice versa. Stiffness data are given in means ± standard deviation (SD), *n* ≥ 30, fluorophore intensity data are given in means ± SD, *n* ≥ 27. g) Dex‐TAB/HRP precursor microdroplets were produced via emulsification with oil in a microfluidic droplet generator. h) Enzymatically crosslinked Dex‐TAB microgels were ≈20 µm in diameter and characterized by a monodispersed size distribution (i.e., CV < 5%). i) Microgel elasticity could be tuned via the H_2_O_2_ concentration in the feed channel of the microfluidic crosslinker device. Data are given in means ± SD, *n* ≥ 31. j) Similar to bulk constructs, Dex‐TAB microgels could be further functionalized with multivalent avidin (or analog neutravidin) and subsequently with a biotinylated molecule of interest, as demonstrated with biotinylated FITC. k) Biotin/avidin/biotin coupling was confirmed by measuring fluorescence recovery after photobleaching (FRAP). Scale bars indicate 20 µm, unless otherwise indicated.

To enable the creation of smart multistimuli responsive microgels, we first engineered an injectable hydrogel that could be chemically and mechanically postmodified using orthogonal and cytocompatible secondary crosslinking strategies. To this end, dextran was selected as a suitable polymer backbone owing to its bio‐inert, cytocompatible, and easily modifiable nature.^[^ ^22^ ^]^ Tyramine and biotin were selected as reactive side groups that could be enzymatically crosslinked and further functionalized via biotin/avidin interaction, respectively,^[^ ^17k^ ^,^ ^23^ ^]^ in a fully orthogonal and cytocompatible manner (**Figure** **1**a). Successful dextran‐tyramine‐biotin (Dex‐TAB) synthesis was confirmed using ^1^H NMR (Figure S1, Supporting Information). Dex‐TAB solution was injectable and in situ crosslinkable following the formation of tyramine‐tyramine bonds using horseradish peroxidase (HRP) and H_2_O_2_ as catalyst and oxidizer, respectively (Figure 1b).

Dex‐TAB hydrogel allowed for independent mechanical and chemical tuning by exploiting unreacted tyramines and biotins, respectively. To demonstrate this, Dex‐TAB solution was injected and crosslinked in a cylinder‐shaped mold using HRP and a substoichiometric amount of H_2_O_2_, which resulted in a shape‐stable hydrogel construct with an elastic modulus (herein also referred to as stiffness) of 12.1 ± 0.7 kPa (Figure 1c). In situ chemical modification was readily achieved by incubating the hydrogel construct consecutively with multivalent biotin‐binding avidin analogs (i.e., neutravidin) and a biotinylated fluorophore (i.e., B‐atto), which supramolecularly complexed with the biotins in the hydrogel (Figure 1d). Introducing neutravidin only had a minor effect on the hydrogel's stiffness (16.5 ± 1.8 kPa) as comparted to the subsequent enzymatic postcure using additional H_2_O_2_ (Figure 1e), which increased the stiffness of the hydrogel by more than twofold (39.3 ± 14.4 kPa) (Figure 1f). The concentration of biotin in the microgels was set to ≈1 × 10^−3^ m, which equated to an interbiotin spacing of ≈12 nm and was assumed sufficiently sparse (i.e., >5 nm) to minimize Dex‐TAB stiffening potentially caused by avidin‐induced crosslinking.^[^ ^24^ ^]^ Furthermore, this degree of biotinylation was designed to be sufficient for presenting integrin binding moieties at densities compatible with cell adhesion, spreading, and focal adhesion formation (Figure S2, Supporting Information). Enzymatic postcuring had no effect on the biotin‐mediated fluorescent labeling, which confirmed the near‐independent tuning of chemical and mechanical properties of Dex‐TAB hydrogels via orthogonal supramolecular and enzymatic crosslinking strategies, respectively.

Dex‐TAB and HRP were injected in a microfluidic droplet generator operated in flow‐focusing mode to produce crosslinkable microdroplets (Figure 1g). These microgel precursor droplets were covalently crosslinked via controlled supplementation of H_2_O_2_ using a diffusion‐based microfluidic crosslinking platform that we have recently developed (Figure S3, Supporting Information).^[^ ^25^ ^]^ This resulted in the formation of monodisperse Dex‐TAB microgels with a diameter of 20.7±0.6 µm (Figure 1g). Microgels’ stiffness could be tuned from 6.9 ± 1.9 kPa (i.e., soft) to 70.5 ± 10.8 kPa (i.e., stiff) by varying the concentration of H_2_O_2_ flown through the diffusion‐based crosslinking platform, which dictated the amount of crosslinker to which Dex‐TAB microdroplets were exposed (Figure 1i). Similar to the bulk hydrogels, Dex‐TAB microgels could be further functionalized with biotinylated fluorophores via supramolecular complexation with neutravidin (Figure 1j; Figure S4, Supporting Information). Confocal fluorescence microscopy and fluorescence recovery after photobleaching (FRAP) confirmed that biotin‐FITC was coupled to Dex‐TAB microgels, but not to nonbiotinylated (i.e., Dex‐TA) microgels, which confirmed the successful generation and reactivity of Dex‐TAB microgels (Figure 1k).

### 2.2 Engineering Composite Microtissues via Integrin‐Binding SCMs

**Figure 2:**
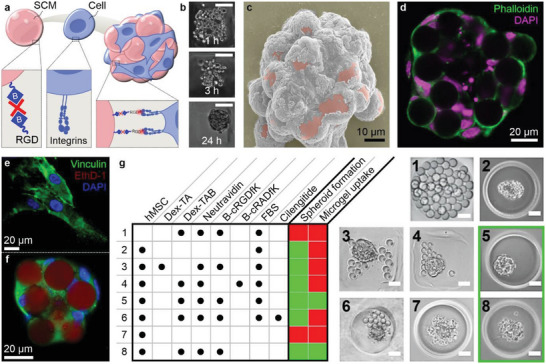
Engineering living composite microtissues using cellular integrin‐binding SCMs. a) Dex‐TAB microgels were functionalized with biotinylated c(RGDfK) peptides via supramolecular complexation with avidin‐type molecules. b) Integrin‐binding c(RGDfK)‐functionalized Dex‐TAB microgels (i.e., SCMs) aggregated with MSCs within 1 day and were imaged using c) scanning electron microscopy (microgels pseudo‐colored pink) and d) confocal fluorescence microscopy (phalloidin in green, DAPI in magenta). e) MSCs cultured atop c(RGDfK)‐functionalized Dex‐TAB hydrogel (i.e., 2D) or f) as a microgel‐laden cell spheroid (i.e., 3D) formed clustered focal adhesions as indicated by positive vinculin staining. g) Coculturing various combinations (indicated with dots) of MSCs, microgels with and without c(RGDfK) functionalization, FBS, and integrin inhibitor Cilengitide revealed that composite microtissue assembly is dependent on RGD‐type cell–material interactions, and that c(RGDfK)‐modified microgels readily enable serum‐free composite 3D tissue formation. Scale bars indicate 100 µm, unless otherwise indicated.

To generate smart/stimuli‐responsive cell‐adhesive microgels (SCMs) that enable self‐assembly of microgels and cells into engineered living composite tissues, we endowed cell‐sized microgels with bio‐adhesive ligands. Specifically, we tethered biotin‐(PEG)_2_‐c(RGDfK) (i.e., a biotinylated cyclic RGD peptide) to Dex‐TAB microgels via supramolecular complexation with neutravidin (**Figure** **2**a). Self‐assembly of mesenchymal stem cells (MSCs) and c(RGDfK)‐functionalized microgels into engineered composite microtissues was achieved within one day after being coseeded in non‐cell‐adhesive microwell arrays (Figure 2b; Figure S5, Supporting Information).^[^ ^26^ ^]^ Scanning electron microscopy and confocal fluorescence microscopy revealed that SCMs were homogeneously distributed throughout the microtissues indicating an appropriate balance between the potential cell–cell and cell–SCM binding energies (Figure 2c,d). Live/dead staining of the composite microtissues confirmed that Dex‐TAB microgels, as well as the self‐assembly process, were cytocompatible (Figure S6, Supporting Information).

Supramolecular complexation of biotinylated RGD moieties to Dex‐TAB using neutravidin consequently resulted in RGD spacing of approximately 12 nm (i.e., similar to the spacing of biotins in Dex‐TAB), which supported cell spreading and focal adhesion formation when cultured in 2D (i.e., atop hydrogel disks) as indicated by the characteristic speckled vinculin pattern (Figure 2e; Figure S2, Supporting Information).^[^ ^27^ ^]^ In composite microtissues, vinculin staining was also positive, albeit slightly more homogeneous and with less visible clusters than in 2D (Figure 2f), which is in line with literature.^[^ ^28^ ^]^ Qualitative assessment of composite tissue spheroid assembly and microgel uptake of different cell/microgel combinations revealed that the interaction between MSCs and SCMs was specifically mediated via the RGD‐type integrins (Figure 2g; Figure S7, Supporting Information). Only microgels functionalized with c(RGDfK) peptides, but not those functionalized with c(RADfK) peptides, could facilitate cell/microgel assembly, while those treated with the soluble cyclic RGD peptide Cilengitide (i.e., an integrin *α*_v_ *β*_3_ and *α*_v_ *β*_5_ inhibitor)^[^ ^29^ ^]^ fully prevented microgel uptake by cell spheroids (Figure 2g; Figure S8, Supporting Information). Cell and SCMs with 1 × 10^−3^ m RGD consistently failed to form cell/microgel spheroids in the absence of fetal bovine serum (FBS), which contains several proteins with RGD sequences (e.g., vitronectin and fibronectin) that are known to drive cell attachment and aggregation (Figure S9, Supporting Information).^[^ ^30^ ^]^ Interestingly, increasing the c(RGDfK) concentration in SCMs from 1 × 10^−3^ to 12 × 10^−3^ m and thereby reducing the inter‐RGD spacing by more than twofold (from ≈12 to ≈5 nm) rescued the self‐assembly of MSCs and SCMs under serum‐free culturing conditions (Figure 2g; Figure S10, Supporting Information), which indicated that RGD‐type cell–material interactions possess the potential to solely steer composite tissue self‐assembly under chemically defined conditions. This feature has vast potential to drive clinical and industrial application of engineered microtissues as it can obviate the use of sera, which are commonly associated with batch‐to‐batch variations and risk of xenogeneic pathogens.^[^ ^31^ ^]^

### 2.3 Programming Lineage Commitment of 3D MSC Spheroids via SCM Elasticity

**Figure 3:**
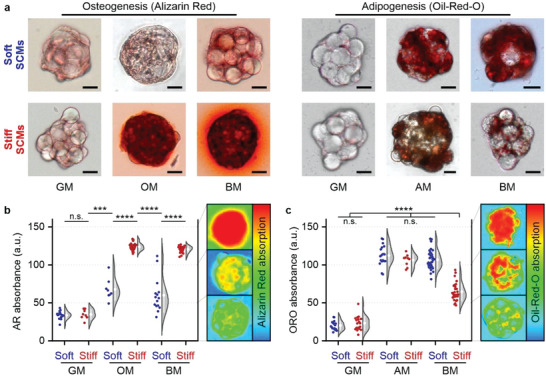
Programming of lineage commitment in 3D MSC spheroids via SCM elasticity. a) Alizarin Red (AR) and Oil‐Red‐O (ORO) staining of composite microtissues containing soft versus stiff SCMs that have been cultured for three weeks in growth medium (GM), osteogenic medium (OM), or bipotential medium (BM). b,c) Representative heat plots and quantification of the per‐spheroid d&nbsp;AR b) and ORO c) staining of calcified matrix and lipid deposition as markers for composite microtissues’ osteogenic and adipogenic differentiation, respectively. AR b) and ORO c) absorbance data are presented as raw datapoints (left) and a Kernel density estimation using Scott's bandwidth smoothing with superimposed means ± SD (right), *n* ≥ 8, significance is indicated (****p < 0.0001, ***p < 0.001, “n.s.” *p* > 0.05, Mann–Whitney). Scale bars indicate 20 µm.

**Figure 4:**
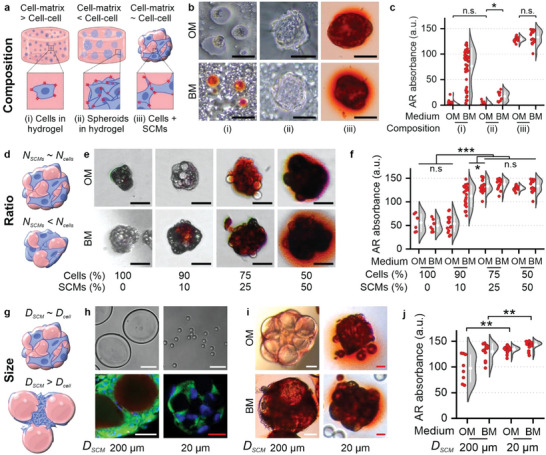
Optimizing cell–material interactions controls composite microtissue fate. a,b) Various 3D composite tissue designs comprising MSCs and stiff c(RGDfK)‐modified Dex‐TAB hydrogel were evaluated for their osteogenic potential using alizarin red staining (AR) after three weeks of being cultured in OM and BM. c) Quantification of per‐cell and per‐spheroid AR staining as a marker for osteogenic differentiation. d–f) Osteogenic differentiation of composite microtissues as a function of the relative number of stiff cell‐sized SCMs (*N*_SCMs_) and MSCs (*N*_cells_). g,h) Composite microtissues containing cell‐sized (*D*_SCM_ ≈ 20 µm) and larger (*D*_SCM_ ≈ 200 µm) stiff SCMs were made. Confocal microscopic analysis of samples stained with vinculin (green) and DAPI (blue) confirmed homogeneous distribution of SCMs throughout the composite microtissues. i,j) Representative microphotographs and quantification of the per‐spheroid AR staining as a marker for osteogenic differentiation of composite microtissues cultured in OM and BM. AR absorbance data c,f,j) are presented as raw datapoints (left) and a Kernel density estimation using Scott's bandwidth smoothing with superimposed means ± SD (right), *n* ≥ 6, in (c) significance between all conditions is ****p < 0.0001, unless otherwise indicated, in (f,j) significance is indicated (***p < 0.001, **p < 0.01, *p < 0.05, “n.s.” *p* > 0.05, Mann–Whitney). Scale bars indicate 50 µm (black), 100 µm (white), and 20 µm (red).

A cell's microenvironmental elasticity can be harnessed to control migration, proliferation, apoptosis, metabolism, and differentiation.^[^ ^9^, ^32^ ^]^ To this end, we produced SCMs with an elastic modulus of 6.9 ± 1.9 kPa (i.e., soft) and 70.5 ± 10.8 kPa (i.e., stiff), which corresponded to the native tissue microelasticities of relatively soft tissues such as lung and muscle (≈5–15 kPa) and pericellular niches within stiff tissues such as precalcified bone (≳40 kPa), respectively.^[^ ^33^ ^]^ Self‐assembly of mechanically pre‐defined SCMs with MSCs in equal number ratios (50:50) readily enabled stiffness‐controlled lineage programming of differentiating stem cells (**Figure** **3**a–c). This result is remarkable, as cellular spheroids without SCMs and containing only MSCs that were cultured under the same conditions could not induce any detectable osteogenic differentiation (see also **Figure** **4**e). Furthermore, SCM stiffness‐dependent osteogenic differentiation of stem cells within composite microtissues was observed when cultured in osteogenic medium (OM) for three weeks, which revealed significantly more calcified matrix (i.e., Alizarin Red (AR) staining) in tissues with stiff SCMs as compared to soft ones. In contrast, SCM stiffness did not have a significant effect on the adipogenic differentiation potential of composite microtissues when cultured in adipogenic medium (AM), as shown by equal intracellular lipid deposition (i.e., Oil‐Red‐O (ORO) staining). Regardless, mechanically controlled MSC programming was confirmed in a chemically unbiased manner by culturing composite microtissues in bipotential differentiation medium (BM) consisting of a balanced mixture of OM and AM. Indeed, lineage commitment of composite microtissues in BM was predominantly dictated by the SCM stiffness, where soft and stiff microgels correlated to adipogenic and osteogenic phenotypes, respectively. While previously published work suggested that osteogenesis in microgel/MSC composite tissues was improved via oxygen diffusion, our work indicates that this action is related to integrin‐mediated mechanical interactions, as the oxygen diffusion through soft and stiff SCMs is presumed similar.^[^ ^15c^ ^]^

### 2.4 Optimizing Cell–Material Interactions Controls Composite Microtissue Fate

Stem cells respond to both cell–cell interactions (e.g., intercellular tension, gap‐junction communication, and plasma membrane receptor stimulation) and cell–biomaterial interactions (e.g., material remodeling, stress relaxation, elasticity, and topography). However, it is not trivial to present such matrix‐type cues within and throughout the volume of cellular spheroids as they are mainly assembled via cell–cell interactions (e.g., cadherins) instead of cell–matrix interactions (e.g., integrins).^[^ ^7^ ^]^ Consequently, cellular microtissues composed of only cells can even become unsensitive to biophysical cues from their surrounding matrix.^[^ ^21^ ^]^

We postulated that the balance between cell–cell and cell–SCM interactions is key to material‐controlled cell programming. We therefore aimed to identify and optimize the biophysical parameters that control cellular behavior in composite microtissues. Based on our previous result showing that SCM stiffness played a dominant role in osteogenic differentiation of MSCs, the following experiments focused on osteogenesis and were performed using c(RGDfK)‐functionalized stiff Dex‐TAB hydrogels and osteogenic or bipotential culture media unless otherwise indicated. To study the effect of cell–biomaterial composition on osteogenesis, cells were i) individually embedded in hydrogel, ii) embedded as cell spheroids in hydrogel, and iii) self‐assembled with SCMs into composite microtissues (Figure 4a). Individual MSCs in hydrogel could differentiate towards the osteogenic lineage, which corresponded to previous studies focusing on stiffness‐controlled differentiation of MSCs in hydrogels (Figure 4b).^[^ ^8^, ^34^ ^]^ Cell‐only spheroids embedded in hydrogel were characterized by negligible levels of osteogenesis, which corroborated a previous study that reported on the encapsulation of MSC spheroids in soft versus stiff hydrogels.^[^ ^21^ ^]^ Interestingly, engineering composite microtissues where stiff SCMs are located in between MSCs not only fully rescued the osteogenic differentiation, but demonstrated a calcification level that significantly surpassed the conventional cells‐in‐hydrogel composition (Figure 4c). This suggested that balancing cell–cell and cell–material interactions is of high value for stem cell lineage programming and SCMs can be used as an instrumental tool to engineer this balance within living 3D tissues.

The cell–cell to cell–matrix interaction balance can potentially be tuned by controlling the amount and/or size of the SCMs. We therefore investigated the effect of cell‐to‐SCM number ratio to reveal the relative amount of SCMs required to mechanically program composite microtissues (Figure 4d). We identified that a minimum of 10% SCMs was needed to exert a notable osteogenic effect on MSC spheroids, and further increasing the amount of SMCs per microtissues associated with an increased amount of osteogenesis defined as microtissue calcification (Figure 4e,f). Aggregating MSCs with an equal amount of soft SCMs did not result in an osteogenic phenotype (see also Figure 3a,b), which corroborated our hypothesis that the fate of cellular microtissues could be effectively steered via SCM‐based biomechanical cues, the level of which depended on the relative number of SCMs within a composite microtissue.

We next investigated whether osteogenic differentiation of MSCs in composite microtissues was affected by SCM size (Figure 4g). Specifically, we postulated that SCMs that are significantly larger than cells would create a composite microtissue architecture that is defined by substantial interstitial voids, which could host multiple cells resulting in local dense cell areas and thus relatively more cell–cell interactions and less cell–material interactions. In theory, random dense packing of microgels with a diameter of ≈20 µm) will provide interstitial void volumes of ≈3 pL, thus offering space for approximately a single MSC (with cell diameter ≈18 µm), while 200 µm SCMs associate with interstitial void volumes that are three orders of magnitude larger and could easily house dozens to hundreds of cells (Figure S11, Supporting Information). Indeed, composite microtissues with stiff SCMs of ≈200 µm contained interstitial voids housing numerous cells (Figure 4h), which associated with significantly less osteogenic differentiation as compared to that observed in composite microtissues containing 20 µm SCMs of the same stiffness and volume fraction, especially when cultured in OM (Figure 4i,j). Of note, microgels much smaller than cells were not considered, as these were prone to cellular internalization and phagocytosis.^[^ ^18^, ^35^ ^]^ These data demonstrated that varying SCM amount and size can be leveraged to control composite microtissue design, which effectively granted control over the lineage commitment of 3D stem cell spheroids.

This set of experiments revealed significantly less or even absence of osteogenic differentiation when stem cells were predominantly interacting with each other or with the surrounding hydrogel as compared to balanced exposure to cell–cell and cell–material interactions such as within composite microtissues. The suboptimal osteogenesis was particularly noticed when culturing unbalanced cell/material constructs in osteogenic medium, but culturing these constructs in bipotential medium could (partly) rescue osteogenesis (Figure 4; Figure S12, Supporting Information). We attributed increased osteogenesis to the higher concentration of the chemical osteogenic differentiation inducer dexamethasone.^[^ ^36^ ^]^ When cell–cell and cell–material interactions were balanced (i.e., composite microtissues containing a 50% number ratio of MSCs and stiff SCMs) osteogenic differentiation occurred equally efficient in both osteogenic and bipotential differentiation media. Together, these findings demonstrated the importance of balanced cell–cell and cell–material interaction for material stiffness‐controlled lineage decision in differentiating stem cells.

### 2.5 On‐Demand In Situ SCM Stiffening Offers Temporal Control over Composite 3D Microtissues

**Figure 5:**
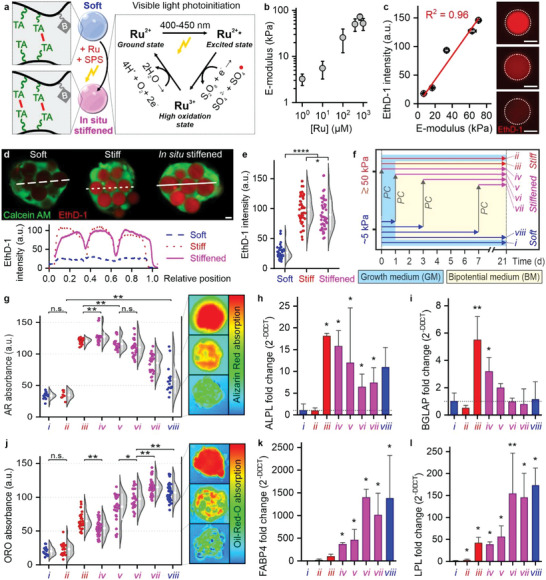
In situ tuning of the biophysical properties of composite microtissues. a) Schematic representation of light‐induced stiffening of soft SCMs using Ru/SPS. Visible light photoinitiation scheme. b) Stiffness could be tuned via the ruthenium concentration. Stiffness data are given in means ± SD, *n* ≥ 30. c) EthD‐1 fluorescence intensity linearly correlated with the stiffness of the Dex‐TAB microgels. Data are given in means ± standard error, *n* ≥ 35 (fluorescence intensity), *n* ≥ 31 (stiffness). d) Confocal fluorescence microscopy was used to visualize and e) quantify the stiffness of soft, as‐prepared stiff, and in situ stiffened SCMs within composite microtissues. EthD‐1 intensity data are presented as raw datapoints (left) and a Kernel density estimation using Scott's bandwidth smoothing with superimposed means ± SD (right), *n* ≥ 46, significance is indicated (****p < 0.0001, *p < 0.05, Mann–Whitney). f) Experimental design to determine the effect of timing in situ SCM stiffening on lineage programing of differentiating MSCs in composite microtissues. Different experimental conditions are indicated with Roman numerals (*i–viii*). g) Representative heat plots and quantification of the per‐spheroid AR staining of calcified matrix deposition, h) BGLAP gene expression, and i) ALPL gene expression as a function of in situ stiffening timing. j) Representative heat plots and quantification of the per‐spheroid ORO staining of intracellular lipid deposition, k) FABP4 gene expression, and l) LPL gene expression as a function of in situ stiffening timing. AR and ORO absorbance data are presented as raw datapoints (left) and a Kernel density estimation using Scott's bandwidth smoothing with superimposed means ± SD (right), *n* ≥ 8 (g), *n* ≥ 17 j), significance between all conditions is ****p < 0.0001, unless otherwise indicated (**p < 0.01, *p < 0.05, “n.s.” *p* > 0.05, Mann–Whitney. Gene expression data h,i,k,l) are given in fold change means ± SD normalized to condition *i* (dotted line, not visible in k and l), *n* = 3, significance compared to condition *i* is indicated (**p < 0.01, *p < 0.05, paired sample *t*‐tests on ΔCT values). Scale bars indicate 10 µm.

The mechanical properties of native tissues change during development, aging, and disease.^[^ ^37^ ^]^ To enable the studying of altered mechanical environments within microtissues, we created SCMs that possess on‐demand tunable mechanical properties. Specifically, we harnessed visible‐light‐induced photo‐crosslinking of the remaining free tyramines in soft Dex‐TAB microgels using a ruthenium complex and sodium persulfate (Ru/SPS) as initiators^[^ ^19^ ^]^ to achieve in situ microgel stiffening (**Figure** **5**a). Photo‐initiated postcuring of SCMs could be controlled via the ruthenium complex concentration, thereby endowing SCMs with the capacity to predictably acquire a well‐defined physiological stiffness following exposure to a noninvasive visible light‐based trigger (Figure 5b). In line with literature,^[^ ^8f^ ^]^ hydrogel stiffness linearly (*R* ^2^ = 0.96) correlated with EthD‐1 staining intensity (Figure 5c). This allowed for noninvasive visualization and quantification of SCM stiffness within composite microtissues before and after visible‐light‐induced postcuring at high spatial resolution. Confocal fluorescence microscopy confirmed that soft SCMs in composite microtissues could be homogeneously stiffened via in situ Ru/SPS‐mediated photo‐crosslinking (Figure 5d), and that on‐demand in situ stiffened and as‐prepared stiff SCMs possessed near‐identical stiffness (Figure 5e).

That biomaterial stiffness can dictate cell behavior is well documented, however, little is known regarding on what timescale biomaterial stiffness is capable of steering cell behavior (e.g., stem cell lineage commitment). We stiffened SCMs within composite microtissues on predefined timepoints to unravel the temporal relevance of biomaterial stiffness on osteogenic lineage commitment of stem cells (Figure 5f). Notably, stiffness‐controlled early‐onset lineage commitment had been reported for MSCs in low cell–cell contact culture systems (e.g., atop 2D hydrogel substrates^[^ ^17e^ ^]^ or within hydrogels),^[^ ^8f^ ^,^ ^17l^ ^,^ ^38^ ^]^ but high cell–cell contact culture systems (e.g., microtissues) have remained unstudied. Stiffening SCMs within composite microtissues after 0, 1, 3, or 7 days of culture in BM resulted in a gradual and significant increase in adipogenesis and decrease in osteogenesis (Figure 5g–l). Dextran‐based microgels that were cultured in the presence of MSCs for 7 days did not reveal significant changes in hydrogel elasticity (Figure S13, Supporting Information), indicating that the SCMs were stable and mechanical cues were not temporarily altered by, for example, hydrogel degradation. Together, our data confirmed the pivotal importance of well‐timed biomechanical cues for controlling cell behavior, which can be straightforwardly imposed throughout engineered tissues using in situ postcurable SCMs.

### 2.6 In Situ Tuning the Biochemical Properties of Composite Microtissues

**Figure 6:**
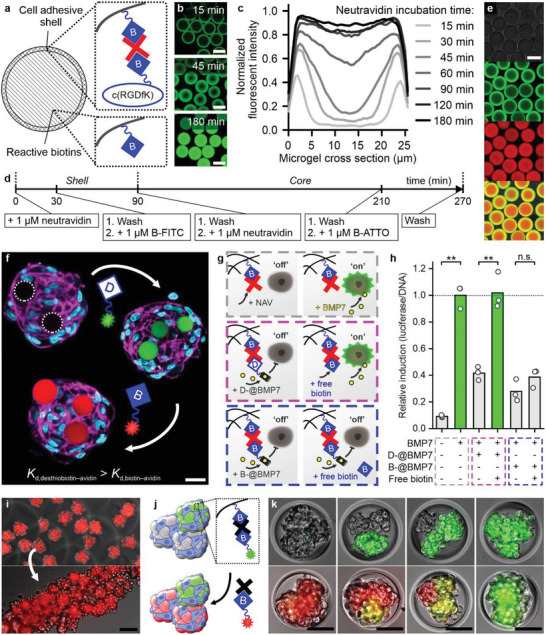
Temporally controlled in situ tuning of the biochemical properties of composite microtissues via supramolecular displacement. a) Schematic representation of an SCM with reactive biotin core. b) Fluorescence confocal microphotographs and c) quantification of spatial fluorescence of Dex‐TAB microgels functionalized with a shell of neutravidin and biotin‐FITC showing that shell thickness is tunable by controlling neutravidin incubation time. d,e) Repeating the supramolecular complexation strategy multiple times with different neutravidin incubation times and biotinylated moieties (biotin‐FITC in green and biotin‐atto565 in red) enabled the formation of multicolored core–shell microgels. f) Confocal fluorescence image of the assembly and in situ biochemical modification of a composite microtissue via the competitive complexation of neutravidin with desthiobiotinylated and biotinylated moieties, as demonstrated by displacing desthiobiotin‐FITC (i.e., green) with biotin‐atto565 (i.e., red). g) SCMs were assembled with a BMP reporter cell line to study in situ biochemical tuning of cell behavior, h) which was quantified via a luciferase assay. Relative induction data are given in means ± SD normalized to the positive control (dotted line), *n* = 3, significance is indicated (**p < 0.01, “n.s.” *p* > 0.05, two‐sample *t*‐test). i) Multiple preformed composite microtissues could be combined to form a larger, for example, line‐shaped modular tissue. j,k) Combining various composite microtissues in different ratios readily enabled their self‐assembly into a variety of larger smart modular microtissues with spatiotemporally controllable microenvironments as demonstrated via postfunctionalization of SCMs with biotinylated fluorophores. Scale bars indicate 20 µm (white) and 100 µm (black).

Assembly of microtissues into larger hierarchically designed tissues is a promising bottom‐up strategy to engineer modular tissues. On‐demand tunable composite microtissues thus represent interesting living microbuilding blocks to engineer modular tissues with the ability to reversibly and sequentially present specific chemical and mechanical properties in a temporally controllable manner. To achieve this, SCMs composed of Dex‐TAB were endowed with a core–shell pattern in which the shell was decorated with cell binding moieties, while the core contained free biotins reactive for subsequent on‐demand functionalization (**Figure** **6**a). By tuning the concentration and incubation time of neutravidin, we could reproducibly control neutravidin's penetration depth into the SCMs. This strategy granted conformal 2.5D control over the microgel's biochemical composition by determining the thickness of the neutravidin‐modified shell, which acted as the reactive substrate for subsequent coupling of biotinylated molecules. Diffusion‐based spatial templating was confirmed and quantified using biotin‐FITC (i.e., green) labeling in combination with confocal fluorescence imaging (Figure 6b,c; Figure S14, Supporting Information). After shell functionalization, the core of the microgels still contained free biotins that could be endowed with another moiety by repeating the functionalization protocol with a prolonged neutravidin incubation step. For example, core–shell multifunctional microgels could be readily prepared by applying a shell functionalization using the multistep functionalization protocol as described in Figure 6d. After confirming the effectiveness of core–shell functionalization protocol using green and red biotinylated fluorophores (Figure 6e), the same core–shell functionalization strategy was used to create temporally reactive SCMs by permanently endowing the microgels’ shells with c(RGDfK) peptides to enable integrin‐mediated cell adhesion and promote self‐assembly, while leaving the cores with reactive biotins available for subsequent on‐demand modifications via biotin/avidin‐type complexation. We previously demonstrated significant permeability of fluorescently labeled molecules with hydrodynamic radii up to several nanometers into Dex‐TAB hydrogels,^[^ ^20^ ^]^ which indicates the flexibility of the SCM postfunctionalization approach.

Composite microtissues were produced using biotinylated SCMs, which acted as templates for temporally controlled sequential presentation of biochemical moieties. To this end, we leveraged the competitive supramolecular complexation of avidin and biotin analogs.^[^ ^20^ ^]^ In short, the dissociation constant (*K*_d_) of desthiobiotin–avidin complexes is significantly higher as compared to those of biotin‐avidin complexes, which enables swift and cytocompatible supramolecular displacement of desthiobiotinylated moieties by biotinylated moieties (Figure S15, Supporting Information). After self‐assembly of composite microtissues, incorporated SCMs where temporally tunable as demonstrated by sequential endowment with desthiobiotin‐FITC (i.e., green) and competitive displacement with biotin‐atto565 (i.e., red). Confocal fluorescence microscopic imaging of composite microtissues confirmed the in situ coupling of desthiobiotin and its displacement by biotin, and thus confirmed successful reversible and sequential functionalization of man‐made living microtissues (Figure 6f).

To proof the concept of on‐demand in situ biochemical programming of microtissues, SCMs were self‐assembled with a bone morphogenetic protein (BMP) reporter cell line (Figure S16, Supporting Information).^[^ ^39^ ^]^ Exposing the resulting composite microtissues to BMP7 indeed triggered the cells to produce significant amounts of bioluminescent luciferase (Figure 6g,h; grey dashed boxes). Subsequent in situ modification of SCMs via supramolecular complexation with neutravidin and a desthiobiotinylated BMP7 neutralizing antibody (D‐@BMP7)^[^ ^40^ ^]^ effectively sequestered BMP7 from the cells as confirmed by the strongly diminished bioluminescence levels. Subsequent addition of free biotin fully reinstated the BMP7‐induced bioluminescence, which corroborated in situ supramolecular displacement of D‐@BMP7 by biotin in the composite microtissues (Figure 6g,h; magenta dashed boxes). In contrast, endowing SCMs with biotinylated BMP7 neutralizing antibodies (B‐@BMP7) also diminished the cellular response to BMP7, but could not be restored by adding free biotins (Figure 6g,h; blue dashed boxes). This confirmed that programming of the temporal behavior of composite microtissues was specifically controlled by on‐demand in situ desthiobiotin/biotin displacement in the SCMs.

A potential application of the composite microtissues lies in the field of bottom‐up or modular tissue engineering, where they could be leveraged as living building blocks for the creation of larger smart modular tissues. Interestingly, modular tissues typically contain intrinsic porosity that mitigates starvation‐induced cell death by improving diffusion of nutrients and waste products, as well as promoting vascularization.^[^ ^14^, ^41^ ^]^ Furthermore, incorporation of micromaterials such as our SCMs into artificial tissue constructs represents an elegant approach to obtain larger grafts using fewer donor cells.^[^ ^10^, ^14^, ^42^ ^]^ We here aimed to provide a proof‐of‐principle of using our composite microtissues as smart modular building blocks for larger tissue engineering. To this end, we first demonstrated that SCM‐based composite microtissues could self‐assemble into larger tissue constructs by straightforwardly combining them into a mold to form, for example, a modular tissue fiber (Figure 6i). To demonstrate the potential of a smart modular tissue design, two variants of composite microtissues were produced containing either biotin‐FITC‐labeled or nonlabeled SCMs. Composite microtissues were seeded at various ratios in non‐cell‐adhesive agarose microwells to form larger modular tissues consisting of three preformed composite microtissues (Figure 6j). The resulting smart modular tissues could be spatiotemporally modified, as demonstrated via the addition of biotin‐atto565, which readily coupled to the SCMs within composite microtissues (Figure 6k). These data confirmed that SCMs can be used to endow larger living modular tissues with smart properties via spatiotemporally controlled bioactive cues.

## 3 Conclusion

In conclusion, we have developed stimuli‐responsive cell‐adhesive micromaterials, in short “SCMs,” that can present biophysically and biochemically tunable cues within 3D cellular spheroids. Specifically, SCMs were composed of a dextran polymer backbone modified with tyramine and biotin moieties, which allowed for in situ crosslinking, postcuring (i.e., biophysical), and postfunctionalization (i.e., biochemical). Furthermore, SCMs were endowed with cyclic RGD peptides to enable autonomous self‐assembly into homogeneous 3D living composite microtissues when cocultured with mammalian cells such as MSCs. Increasing SCM stiffness allowed for efficient and potent osteogenic lineage commitment of differentiating 3D stem cell cultures, which was in sharp contrast to stem cell‐only spheroids that did not demonstrate any notable level of osteogenesis, not even when embedded within stiff 3D bulk hydrogels. Temporally controlled experimentation based on photo‐induced postcuring of SCMs within composite microtissues revealed that material elasticity plays a deciding role in early onset lineage commitment of MSCs. Besides temporal presentation of biophysical cues, SCMs could be on‐demand, reversibly, and sequentially modified to express molecules of interest via a desthiobiotin/biotin supramolecular displacement strategy. This approach enabled the reversible biochemical programming of reporter cells within SCM‐laden composite microtissues. The ability to endow man‐made tissue constructs with in situ tunable biomechanical and biochemical properties through the facile incorporation of SCMs offers novel avenues of research and is anticipated to facilitate further adoption of stem cell technologies in a variety of 3D cell culture applications, including biomolecular, pharmaceutical, and tissue engineering applications by offering control over the microenvironment within engineered living tissues.

## 4 Experimental Section

### Materials

Dextran was purchased from Sigma/Merck (MW 15–25 kg mol^−1^ to M_n_ 16 kg mol^−1^) and functionalized with tyramine and biotin as previously described.^[20, 23b]^ Dextran‐tyramine‐biotin (Dex‐TAB) contained ≈13 and ≈6 tyramine and biotin moieties per 100 repetitive monosaccharide units, respectively. Trifluoroacetic acid (TFA), sodium bicarbonate (NaHCO_3_), biotin‐atto565, biotin‐4‐fluorescein (biotin‐FITC), 6‐aminofluorescein, horseradish peroxidase (HRP, type VI), hydrogen peroxide (H_2_O_2_; with inhibitor), fetal bovine serum (FBS), ascorbic acid, insulin (human), 3‐isobutyl‐1‐methylxanthine (IBMX), indomethacin, dexamethasone, *β*‐glycerol phosphate disodium salt pentahydrate (*β*‐GP), Calcein AM, ethidium homodimer‐1 (EthD‐1), fluorescein isothiocyanate (FITC), Oil Red O, Alizarin Red S, buffered formalin, Triton X‐100, Tween‐20, sodium acetate, sodium azide (NaN_3_), bovine serum albumin (BSA), hexadecane, Span‐80, biotin, antivinculin (V9131), N‐(2‐aminoethyl)maleimide trifluoroacetate salt (amino‐maleimide), primers FABP4 (forward: TCAGTGTGAATGGGGATGTGA; reverse: TCAACGTCCCTTGGCTTATGC), ALPL (forward: ACAAGCACTCCCACTTCATC; reverse: TTCAGCTCGTACTGCATGTC), LPL (forward: ACACAGAGGTAGATATTGGAG, reverse: CTTTTTCTGAGTCTCTCCTG), BGLAP (forward: GGCAGCGAGGTAGTGAAGAG; reverse: GATGTGGTCAGCCAACTCGT), and all solvents (unless otherwise indicated) were purchased from Sigma‐Aldrich/Merck. Neutravidin, 4′,6‐diamidino‐2‐phenylindole (DAPI), agarose (Ultrapure, low melting point, Invitrogen), TRIzol, linear acrylamide, Cryomatrix embedding resin (Shandon), Superfrost Plus Gold adhesion slides, and Alexa Fluor labeled donkey–anti‐mouse secondary antibodies (Invitrogen) were purchased from Thermo Fisher Scientific. Phosphate‐buffered saline (PBS) was purchased from Lonza. Sodium persulfate (SPS) and ruthenium (Ru) were purchased from Advanced BioMatrix. MaxiSorp well plates were purchased from Nunc. Reporter Lysis Buffer (E397A), luciferase assay reagent (E1483), and QuantiFluor dsDNA System were purchased from Promega. Recombinant human BMP7 (354‐BP‐010) was purchased from R&D Systems). Desthiobiotinylated (D‐@BMP7) and biotinylated (B‐@BMP7) V_H_H were synthesized as previously published.^[^ ^20^ ^]^ Chloroform was purchased from WR Chemicals. RNase‐free H_2_O was purchased from Qiagen. iScript cDNA Synthesis Kit was purchased from BioRad. SensiMix SYBR & Fluorescein Kit (QT615‐20) was purchased from Bioline. Minimal Essential Medium *α* with nucleosides (*α*MEM), Dulbecco's modified Eagle's medium (DMEM), penicillin and streptomycin, GlutaMAX, and trypsin–EDTA were purchased from Gibco. Basic fibroblast growth factor (ISOKine bFGF) was purchased from Neuromics. Phalloidin‐AF488 and phalloidin‐AF647 were purchased from Molecular Probes. Biotin‐(PEG)_2_‐c(RGDfK) (PCI‐3697‐PI), biotin‐(PEG)_2_‐c(RADfK) (PCI‐3808‐PI), and c(RGDyK) (PCI‐3662‐PI) were purchased from Peptides International. Cilengitide was purchased from Tocris Bioscience. Catalase (from bovine liver) was purchased from Wako. Polydimethylsiloxane (PDMS, Sylgard 184) was purchased from Dow Corning. Aquapel was purchased from Vulcavite. Pico‐Surf 1 in Novec 7500 Engineered Fluid and Pico‐Break 1 were purchased from Dolomite. Gastight syringes (Hamilton), fluorinated ethylene propylene tubing (FEP, inner diameter 250 µm, DuPont) and connectors were purchased from IDEX Health and Science. Silicone tubing (inner diameter 310 µm, outer diameter 640 µm) was purchased from Helix Medical. Low pressure syringe pumps (neMESYS) were purchased from Cetoni. Surfactant‐free fluorocarbon oil (Novec 7500 Engineered Fluid) was kindly provided by the BIOS Lab‐on‐a‐Chip group (University of Twente). C2C12‐BRE‐Luc cells were kindly provided by prof. Daniel B. Rifkin.

### Hydrogel Disk Production

Dex‐TAB hydrogel with a biotin concentration of 1 × 10^−3^ m were produced by mixing 90 g L^−1^ Dex‐TA, 10 g L^−1^ Dex‐TAB, 3 U mL^−1^ HRP, and 0.3 g L^−1^ H_2_O_2_ in PBS. Hydrogel disks were produced by injection molding the premixed and ice‐cooled components into disk‐shaped PDMS molds with a diameter and thickness of 5 and 2 mm, respectively. Hydrogels were gelled at room temperature for 10 min and then washed three times with PBS.

### Microgel Production

To generate ≈20 µm microgels, a microfluidic mixer, droplet generator, and H_2_O_2_ diffusion‐based crosslinking chips were fabricated with ≈100 × 10^−6^ m, ≈25 µm, and ≈100 µm high channels, respectively, as previously described.^[^ ^25^ ^]^ Aquapel was introduced in the chips before usage to ensure channel wall hydrophobicity. Using FEP tubing, chips were connected to each other and to gastight syringes, which were controlled by low‐pressure syringe pumps. To generate hydrogel precursor microdroplets, PBS with 10% (v/v) Dex‐TA and 40 U mL^−1^ HRP was emulsified in Novec 7500 Engineered Fluid oil containing 2% (w/w) Pico‐Surf 1 surfactant at a 1:6 (hydrogel:oil) flow ratio (2 µL min^−1^ hydrogel, 12 µL min^−1^ oil). The hydrogel precursor microemulsion was flown at a total rate of 14 µL min^−1^ through the connected diffusion platform, which was also fed with H_2_O_2_ flowing in opposite direction at a rate of 30 µL min^−1^. The H_2_O_2_ diffused from the feed channel through the PDMS walls into the gel precursor microemulsion, thereby triggering enzymatic crosslinking of tyramine‐conjugated polymer, as previously described.^[^ ^25^ ^]^ Dex‐TAB microgels with a biotin concentration of 1 × 10^−3^ m were formed using a hydrogel premix containing Dex‐TA and Dex‐TAB in a 9:1 weight ratio (similar to Dex‐TAB bulk hydrogels). Cell‐laden microgels were generated via a similar procedure, except using serum‐free medium with 10% (v/v) Dex‐TA, 40 U mL^−1^ HRP, and 10^6^ MSCs as the hydrogel precursor mixture. The microemulsion was broken by washing three times with surfactant‐free fluorocarbon oil and subsequent supplementation of Pico‐Break 1 in the presence of PBS that contained 0.05% (w/v) NaN_3_ for preservation and 1% (w/v) BSA to prevent microgel aggregation.

To generate ≈200 µm microgels, a microfluidic droplet generator with exchangeable nozzle was used, as previously described.^[^ ^43^ ^]^ Hydrogel precursor solutions were identical to the ones 20 µm microgels production protocol, and emulsified in hexadecane with 1% (v/v) Span‐80 at a 1:6 (hydrogel:oil) flow ratio (10 µL min^−1^ hydrogel, 60 µL min^−1^ oil). To induce crosslinking, the microemulsion was flown through a semipermeable silicone tubing that was submerged in a bath of 300 g L^−1^ H_2_O_2_, collected in an oil phase that consisted of H_2_O_2_‐laden crosslinker emulsion prepared as previously described,^[^ ^44^ ^]^ and incubated for 3 h on a rollers mixer to ensure complete crosslinking of the microgels. The microgel emulsion was broken by washing three to five times with surfactant‐free hexadecane followed by addition of PBS that contained 0.05% (w/v) NaN_3_ for preservation and 1% (w/v) BSA to prevent microgel aggregation.

On‐chip droplets were visualized using a stereomicroscope set‐up (Nikon SMZ800 equipped with Leica DFC300 FX camera). Retrieved microgels were imaged using phase contrast microscopy and the size distribution was measured using Matlab software.

### Dex‐TAB Functionalization

To functionalize Dex‐TAB hydrogel disks with biotinylated moieties, they were incubated 4 h with 1 × 10^−6^ m neutravidin in PBS, washed three times for 10 min with PBS, incubated for 4 h with 1 × 10^−6^ m of the biotinylated molecule of interest, and washed again three times for 10 min with PBS. To functionalize Dex‐TAB microgels with biotinylated moieties, they were washed three times with excessive washing buffer that consisted of 10 g L^−1^ BSA in PBS to remove NaN_3_, consecutively incubated with neutravidin in washing buffer, washed with washing buffer, incubated with a biotinylated or desthiobiotinylated molecule of interest, and washed again with washing buffer, as further specified in the results section. If necessary, the functionalization protocol was repeated, for example, to create core–shell functionalized microgels.

For fluorescence microscopy (EVOS FL), confocal fluorescence microscopy (Zeiss LSM 510 and Nikon A1+), and fluorescence recovery after photobleaching (FRAP; Zeiss LSM 510), hydrogels were functionalized with biotin‐atto565, biotin‐FITC, and/or desthiobiotin‐FITC. The FRAP curve was obtained by plotting, as a function of time, the fluorescent intensity of the bleach spot minus the background normalized for the bleach‐rate corrected average intensity before bleaching, where the bleach rate was determined by normalizing the sample's fluorescent intensity besides the bleach spot normalized for its average intensity before bleaching, as previously described.^[^ ^20^ ^]^

To enable cell adhesion, hydrogels were functionalized with cyclic RGD peptides. To this end, the hydrogels were incubated for 30 min with 1 × 10^−6^ m neutravidin in washing buffer (see Section “Dex‐TAB functionalization”), washed with washing buffer, and subsequently incubated for 60 min with 1 × 10^−6^ m biotinylated cyclic RGD peptide biotin‐(PEG)_2_‐c(RGDfK) in washing buffer, as also depicted in Figure 6d. Dex‐TA (i.e., without biotin) microgels that had been treated with the same functionalization protocol and Dex‐TAB microgels that had been functionalized with biotin‐(PEG)_2_‐c(RADfK) were used as controls. To endow Dex‐TAB bulk gels with an equivalent concentration (≈1 × 10^−3^ m) of RGD moieties, tyramine‐modified c(RGDyK) was homogeneously mixed and enzymatically crosslinked in the Dex‐TAB hydrogel.

### In Situ Stiffening

Hydrogel disks and microgels were stiffened by incubating them with 40 U mL^−1^ HRP for 30 min after which H_2_O_2_ was added to a final concentration of 0.3 g L^−1^. When cell were present, the enzymatic postcure was terminated by adding bovine catalase to a final concentration of 6 kU after 90 s, which immediately consumed all remaining H_2_O_2_ through a competitive enzymatic reaction.^[^ ^44^ ^]^ Alternatively, microgels were stiffened by incubating them with 2.5 × 10^−3^ m SPS for 30 min, after which ruthenium complex was added to a final concentration of 1 × 10^−6^ to 4 × 10^−3^ m (depending on the experiment). Free‐radical crosslinking was induced using 15–180 s (depending on the experiment) irradiation with visible light (white LED, ≈30 mW cm^−2^).

### Mechanical Characterization

Hydrogels were mechanically characterized using nanoindentation (Optics11, Pavone). Indentation measurements were performed in PBS using a cantilever (spring constant 0.27 N m^−1^) with a glass colloidal probe (radius = 7.5 µm) attached to the tip. The probe was brought into close contact with the surface of the micro‐ or macrogel and indentation was performed using the following indentation procedure: an indentation of 500–2000 nm (depending on the size of the gel) for 2 s followed by a 1 s holding and 2 s retraction time. To minimize movement of microgels during nanoindentation, they were seeded into a MaxiSorp 96‐well plate. The obtained indentation curves were fitted between 0 and 500 nm indentation using the Hertzian model from which the effective elastic modulus was obtained, according to *P* = 4/3 *E*_eff_ *R* ^1/2^ *h* ^2/3^, where *P* is the applied load, *E*_eff_ is the effective Young's Modulus, *R* is the radius of the indentation tip, and *h* is the indentation depth. The elastic modulus (herein also referred to as stiffness) was calculated by *E* = *E*_eff_(1 – *ν* ^2^), assuming a Poisson's ratio of *ν* = 0.5.

### Cell Isolation and Expansion

Human mesenchymal stem/stromal cells (MSCs) were isolated from fresh bone marrow samples and cultured as previously described.^[^ ^45^ ^]^ The use of patient material was approved by the local ethical committee of the Medisch Spectrum Twente and informed written consent was obtained for all samples (METC∖06003). In short, nucleated cells in the bone marrow aspirates were counted, seeded in tissue culture flasks at a density of 500 000 cells cm^−2^ and cultured in MSC growth medium, consisting of 10% (v/v) FBS, 100 U mL^−1^ penicillin, 100 mg L^−1^ streptomycin, 1% (v/v) GlutaMAX, 0.2 mm ascorbic acid, and 1 µg L^−1^ bFGF (added fresh) in *α*MEM.

C2C12‐BRE‐Luc cells were cultured in growth medium consisting of 20% (v/v) FBS, 1% (v/v), 100 U mL^−1^ penicillin, and 100 mg L^−1^ streptomycin in DMEM. Cells were cultured under 5% CO_2_ at 37 °C and medium was replaced two times per week. When cell culture reached near confluence, the cells were detached using 0.25% (v/v) Trypsin‐EDTA at 37 °C and subsequently subcultured or used for experimentation.

For 2D cultures on hydrogel disks, cells were seeded at a density of 2000 cells cm^−2^. For 3D cultures in bulk hydrogels, cells or cell spheroids were seeded at a density of 2×10^6^ cells cm^−3^. Cell/microgel spheroid formation is described below.

### Composite Microtissue Formation, Microgel Uptake, and Inhibition

Cells and/or microgels were coseeded into nonadherent microwell chips that were produced by casting 30 g L^−1^ sterile agarose in demineralized water on an in‐house fabricated mold, as previously described.^[26a]^ For ≈20 µm microgels, the cells and/or microgels were homogenously seeded into agarose constructs containing 3000 microwells (≈200 × 200 × 200 µm cubical‐shaped or 200 × 200 µm column‐shaped) at a seeding density of 50 units (i.e., cells, microgels, or cells + microgels) per microwell (i.e., cells:microgels volume ratio ≈1). For the ≈200 µm microgels, cells and microgels were homogenously seeded into agarose constructs containing ≈1000 × 1000 µm column‐shaped microwells at a cells:microgels number ratio ≈1000:1 (i.e., cells:microgels volume ratio ≈1:1). The cells and/or microgels were allowed to self‐assemble into composite microtissues overnight in growth medium and were subsequently used for further experimentation or washed with PBS and fixated with 10% neutrally buffered formalin.

Cell/microgel spheroid formation and microgel uptake were evaluated using MSCs and C2C12‐BRE‐Luc cells. To study the effect of FBS concentration on cell and cell/microgel spheroid formation, C2C12‐BRE‐Luc cells were coseeded and cultured overnight as described above, but using growth medium with different FBS concentrations as indicated in the results section. Furthermore, the effect of microgels’ c(RGDfK) concentration on microgel uptake was evaluated by using Dex‐TAB microgels with 0, 1 × 10^−3^, and 12 × 10^−3^ m c(RGDfk) in the shell, achieved by using different Dex‐TA:Dex‐TAB ratios during microgel production. Spheroid formation inhibition was studied by adding 0.01 × 10^−3^, 0.1 × 10^−3^, and 1 × 10^−3^ m Cilengitide to the growth medium immediately after seeding the cells and microgels in the microwells. Spheroid formation and microgel uptake were visually qualified (i.e., successful vs unsuccessful) using fluorescent and/or phase contrast microscopic images. Some samples were semi‐quantified by analyzing the circularity of spheroids using ImageJ software. Larger multispheroid tissues were formed by coseeding multiple pre‐aggregated spheroids in nonadherent agarose microwells or silicone molds.

### 3D cell Culture in Dex‐TA Bulk Gels

Dex‐TA bulk gel formation was achieved by mixing MSCs or preformed MSC spheroids with 100 g L^−1^ Dex‐TA, 1 × 10^−3^ m c(RGDyK), 3 U mL^−1^ HRP, and 0.3 g L^−1^ H_2_O_2_ in PBS. Components were mixed on ice, and gelled for 15 min at room temperature. Constructs were then cultured in MSC growth or differentiation medium.

### Multilineage Differentiation

Composite microtissues were cultured in MSC growth medium (GM), MSC adipogenic differentiation medium (AM) consisting of 10% (v/v) FBS, 100 U mL^−1^ Penicillin, 100 mg L^−1^ Streptomycin, 1% (v/v) GlutaMAX, 0.2 × 10^−3^ m ascorbic acid, 10 mg L^−1^ insulin, 0.5 × 10^−3^ m IBMX, 200 × 10^−6^ m indomethacin, and 1 × 10^−6^ m dexamethasone (added fresh), MSC osteogenic differentiation medium (OM) consisting of 10% (v/v) FBS, 100 U mL^−1^ Penicillin, 100 mg L^−1^ Streptomycin, 1% (v/v) GlutaMAX, 0.2 × 10^−3^ m ascorbic acid, 10 × 10^−9^ m dexamethasone (added fresh), and 10 × 10^−3^ m *β*‐GP (added fresh) in *α*MEM, or a 1:1 mixture of adipogenic and osteogenic medium called bipotential medium (BM), which were refreshed three times per week. As a negative control, encapsulated MSCs were cultured in GM supplemented with 10 × 10^−9^ m *β*‐GP.

### Staining and Characterization

Cell viability was analyzed by staining with 2 × 10^−6^ m Calcein AM (i.e., live) and 4 × 10^−6^ m EthD‐1 (i.e., dead) in PBS and visualization using fluorescence microscopy. For additional (confocal) fluorescence analyses, samples were permeabilized using 0.1% (v/v) Triton X‐100 and subsequently stained with 2.5 U mL^−1^ phalloidin‐AF488 or phalloidin‐AF647 (45 min), 1 mg L^−1^ DAPI (15 min), and 4 × 10^−6^ m EthD‐1 (30 min) to stain cellular F‐actin, cell nuclei, and crosslinked Dex‐TA(B) polymer, respectively.

For fluorescent immunohistochemical analysis, samples were incubated 15 min with 150 µL permeation/blocking solution consisting of 0.1% (v/v) Triton‐X100 and 30 g L^−1^ BSA in PBS, followed by 45 min incubation with 150 µL blocking solution consisting of 30 g L^−1^ BSA and 0.05% (v/v) Tween‐20 in PBS. Samples were incubated for 1 h at room temperature with 1:400 mouse antivinculin (i.e., primary antibody) in blocking solution, washed three times with blocking solution, incubated for 1 h with 1:400 AF‐488 anti‐mouse (i.e., secondary antibody) in blocking solution in the dark, followed by one wash with blocking solution. Samples were then counterstained using EthD‐1 and DAPI as described above. Samples were washed three times with PBS and subsequently analyzed using fluorescence (EVOS FL) or confocal fluorescence microscopy (Nikon A1+ or Zeiss LSM 880).

Adipogenic differentiation was analyzed by staining samples with a filtered (0.45 µm) 1.8 g L^−1^ Oil Red O in a 2‐propanol/PBS mixture (3:2), visualizing using brightfield microscopy, and quantifying the per‐cell intensity of the inverted green color channel using ImageJ software. Osteogenic differentiation was analyzed by staining samples with a filtered (0.45 µm) 20 g L^−1^ Alizarin Red S in saline dH_2_O, visualizing using fluorescence microscopy, and quantifying the per‐cell intensity of the inverted green color channel using ImageJ software.

For differentiation analysis of individual MSCs and MSC spheroids in bulk hydrogels, constructs were first cryo‐sectioned. To this end, hydrogels were impregnated for approximately 5 h with Cryomatrix, and subsequently snap‐frozen on the cryotome's (Shandon AS620, Thermo Fisher Scientific) cryobar at −60 °C. 7 µm thick sections were transferred to Superfrost Plus Gold adhesion slides and kept in PBS until staining.

For scanning electron microscopy, samples were dehydrated by incubating in gradually increasing ethanol series from 50% to 100% (i.e., absolute ethanol) followed by critical point drying with CO_2_. The dried samples were then gold‐sputtered (Cressington 108auto) and imaged using scanning electron microscopy (JEOL JSM‐IT1000). Photos were pseudo‐colored using CorelDRAW X7 software.

### Calculations

Assuming a homogeneous distribution of moieties through the hydrogel network, the interbiotin spacing in Dex‐TAB approximately equals (*C*_biotin_ × *N*_A_)^−⅓^ × 10^8^ nm, where *C*_biotin_ is given in m. For a biotin concentration *C*_biotin_ = 1 × 10^−3^ m, the interbiotin spacing equals ≈11.8 nm.

The number of cells per void as a function of microgel diameter approximately equals *V*_cell_/*V*_void_, where *V*_cell_ is the cell volume, which was calculated by 4/3*πr*_cell_^3^, with *r*_cell_ as the cell radius assuming spherical cells, and with *V*_void_ as the volume of voids (i.e., interstitial space not occupied by microgels), which was calculated by 4/3*πr*_microgel_^3^/*φ* * (1 − *φ*), with *r*_microgel_ as the microgel radius assuming spherical microgels, and with *φ* as the microgel packing density which was assumed to be 0.6 (i.e., approximated random packing density of spheres).^[^ ^46^ ^]^

### BMP Induction and Inhibition

For BMP7 induction experiments, C2C12‐BRE‐Luc cells were combined with cell‐sized (≈20 µm) SCMs that were shell‐functionalized with biotinylated c(RGDfk) and core‐functionalized in three different ways: i) with only neutravidin, ii) with neutravidin and D‐@BMP7, and iii) with neutravidin and B@BMP7, respectively. After coseeding and overnight culturing cells and SCMs in non‐cell‐adhesive agarose microwells as described above, the composite microtissues were incubated with 1 × 10^−6^ m biotin in PBS for 4 h to allow supramolecular exchange of the (desthio)biotinylated groups with free biotin. The next day, tissues were starved using growth medium containing 0.5% (v/v) FBS for 10–12 h. The composite tissues were subsequently exposed to 100 ng mL^−1^ of BMP7 for 10–15 h. Microtissues were lysed using reporter lysis buffer and a single freeze‐thaw cycle. Luciferase expression was determined using a luciferase assay following manufacturer's protocol and a luminometer (Perkin Elmer, Victor X3). Luciferase expression was normalized to the total DNA content, which was quantified using the QuantiFluor dsDNA System following manufacturer's protocol and a fluorometer (Perkin Elmer, Victor X3). All conditions are schematically depicted in Figure S16 (Supporting Information).

### RNA Isolation and Gene Expression Analysis

To extract total RNA, samples were washed with PBS and resuspended in 1 mL TRIzol, immediately frozen and stored at −80 °C until further processing. After thawing the lysates on ice, 200 µL chloroform was added and shaken vigorously by hand for 15 s. After 5 min incubation at room temperature (for initial phase separation), samples were centrifuged for 15 min at 120 00 *g* and 4 °C. The upper aqueous phase (≈500 µL) was transferred to a new 1.5 mL Eppendorf tube on ice and 50 µL cold sodium acetate was added to a final concentration of 0.3 m, after which the samples where briefly vortexed. 3.15 µL linear acrylamide up to a final concentration of 15 mL L^−1^ and 550 µL ice cold 2‐propanol were added and mix by gentle pipetting. Samples were incubated overnight at −20 °C, thawed on ice, and centrifuged for 30 min at 12 000 *g* and 4 °C. The supernatant was gently removed using gentle pipetting (without disrupting the pellet), after which the pellet was resuspended in 1 mL ice cold 70% (v/v) ethanol. The pellet was reformed by centrifuging for 5 min at 7500 *g* and 4 °C. The supernatant was immediately removed and the pellet was air‐dried for ≈15 min on ice and subsequently dissolved in 20 µL RNase‐free water. RNA concentration and purity were immediately determined using photospectrometry (Nanodrop, ND‐1000). Isolated RNA was stored at −80 °C until further use.

cDNA was synthesized using the iScript cDNA Synthesis Kit following manufacturer's protocol. Briefly, an iScript master‐mix was prepared by mixing four volumes of 5× iScript Reaction Mix with one volume of iScript Reverse Transcriptase. Subsequently, 5 µL of iScript master‐mix was added to 15 µL of RNA suspension, consisting of a volume equivalent to 200 ng of sample RNA mixed with RNase‐free water. The samples were loaded into a thermal cycler (BioRad, T100) using the iScript protocol (5 min at 25 °C, 30 min at 42 °C, 5 min at 85 °C and hold at 4 °C). The synthesized cDNA was stored at −20 °C until further use.

For qPCR analysis, cDNA samples were processed using the SensiMix SYBR & Fluorescein Kit according to manufacturer's protocol. Briefly, cDNA was thawed on ice and diluted to a concentration of 5 ng cDNA per 8 µL. A qPCR master‐mix was prepared by mixing forward primer (20× concentrated), reverse primer (20× concentrated), and SensiMix SYBR and Fluorescein (2× concentrated) in a 1:1:10 ratio. Per well of a 96‐well plate, 8 µL of diluted cDNA was added to 12 µL qPCR master‐mix. The 96‐well plate was sealed to prevent sample evaporation and centrifuged for 1 min at 3000 g and room temperature. The plate was loaded into a qPCR device (BioRad, CFX96) and processed using the following thermal cycle: (i) 10 min at 95 °C (i.e., melting), (ii) 15 s at 95 °C (i.e., melting), (iii) 15 s at 60 °C (i.e., annealing), and (iv) 15 s at 72 °C (i.e., extension). Steps (ii) to (iv) were repeated 40 times. The qPCR process was followed by recording a melting curve from 65 to 95 °C with a rate of 0.2 °C s^−1^. The data was processed using the 2^−ΔΔCT^ method.^[^ ^47^ ^]^

### Statistics and Schematics

Linear regressions and statistical analysis were performed using OriginPro software. Illustrations were created using CorelDRAW X7 and BioRender software.

## Conflict of Interest

The authors declare no conflict of interest.

## Author Contributions

T.K. and N.G.A.W. contributed equally to this work. According to the Contributor Roles Taxonomy (CRediT) system. T.K.: conceptualization, methodology, validation, formal analysis, investigation, data curation, writing—original draft, writing—review and editing, visualization, supervision, and funding acquisition. N.G.A.W.: validation, formal analysis, investigation, writing—review and editing, and visualization. C.K.: validation, investigation, and visualization. Mi.K.: validation, investigation, and visualization. M.B.: investigation. L.L.: investigation. C.J.: investigation. Ma.K.: writing—review and editing, supervision, and funding acquisition. J.L.: conceptualization, methodology, data curation, writing—review and editing, supervision, and funding acquisition.

## Supporting information

Supporting Information

## Data Availability

The data that support the findings of this study are available from the corresponding author upon reasonable request.
