# Supplementary material for: Steering Stem Cell Fate within 3D Living Composite Tissues Using Stimuli‐Responsive Cell‐Adhesive Micromaterials
Source: Adv Sci (Weinh). 2023 Jan 4;10(10):2205487. doi: 10.1002/advs.202205487 (PMC10074101; doi:10.1002/advs.202205487)
Supplement: Supplementary file 1 — Supporting Information [file ADVS-10-2205487-s001.pdf]

Supporting Information

Steering Stem Cell Fate within 3D Living Composite Tissues using Stimuli-responsive Cell-adhesive Micromaterials

Tom Kamperman, Niels G.A. Willemen,* Cindy Kelder, Michelle Koerselman, Malin Becker, Luanda Lins, Castro Johnbosco, Marcel Karperien, and Jeroen Leijten*

* Shared first authorship

Figures

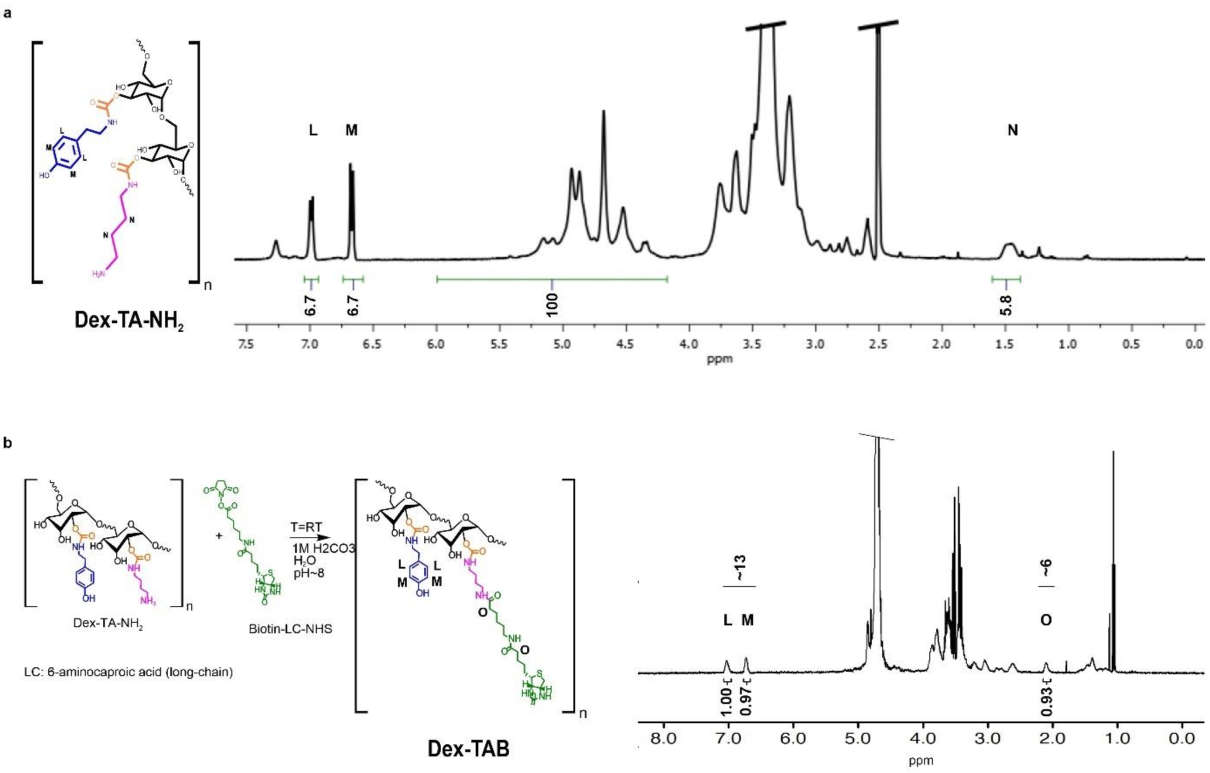

Figure S1. Synthesis and characterization of Dex-TAB. (**a**) Dextran was functionalized with tyramine and butylamine, as previously described.[20, 23b] ^{1}H-NMR analysis was used to confirm the successful synthesis of Dex-TA-NH_2 and to quantify the numbers of conjugated tyramine and butylamine moieties per 100 dextran anhydroglucose rings by calculating the ratios of integrated signals from anomeric and hydroxylic protons of the dextran (δ 4.0 – 5.8 ppm), and aromatic protons of the tyramine groups (δ 6.66 ppm and δ 6.98 ppm) and methyl protons of the butylamine groups (δ 1.4 – 1.5 ppm), respectively. **(b)** Dex-TA-NH_2 was functionalized with biotin using Succinimidyl 6-(biotinamido)hexanoate (biotin-LC-NHS; where LC is 6-aminocaproic acid (long-chain)), as previously described.[20] (**b**) ^{1}H-NMR analysis was used to confirm the successful synthesis of Dex-TAB and to quantify the number of conjugated biotin moieties per 100 dextran anhydroglucose rings, as determined in (a), by calculating the ratio of integrated signals from aromatic protons of the tyramine groups (δ 6.66 ppm and δ 6.98 ppm) and carboxylic amide protons of the coupled 6-aminocaproic spacer (δ 2.13).

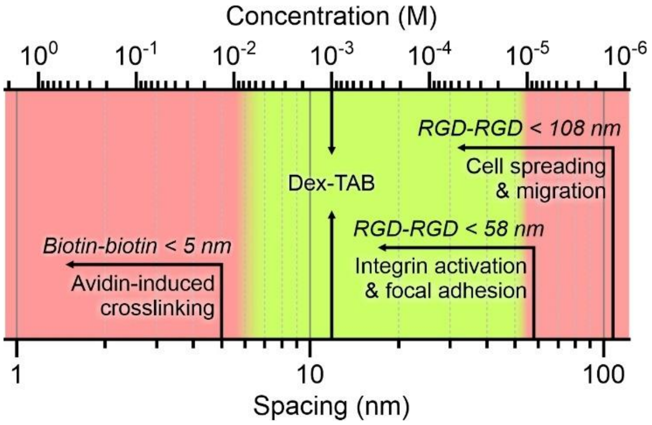

Figure S2. Relation between concentration and spacing of moieties in a 3D material. The 3D inter-molecule spacing was calculated based on the molecular concentration and assuming a homogeneous distribution of moieties through the hydrogel network. For example, the biotin concentration in the Dex-TAB used for most experiments was ~ 1 mM, which corresponds to 12 nm inter-biotin spacing. Biotin concentrations in Dex-TAB > 10 mM would theoretically result in an inter-biotin spacing ≲ 5 nm, which may cause avidin-mediated crosslinking of biotins in Dex-TAB.[24] Biotins can be further functionalized with biotinylated molecules of interest via supramolecular crosslinking with multivalent avidin-type molecules. Functionalizing Dex-TAB that contains 1 mM biotins with biotinylated RGDs using avidin would result in a similar RGD spacing, assuming stochiometric functionalization (i.e., biotin:avidin:biotin = 1:1:1), as previously observed.[20] RGD spacing has been shown to steer cell behavior via interaction with integrins. Specifically, spacing of ≲ 108 nm is needed for cell spreading and migration atop a cell-adhesive biomaterial,[27c] while ≲ 58 nm has been correlated to integrin activation and focal adhesion formation.[27a, 27b] Therefore, biotin concentration in Dex-TAB was tuned to be ~ 1 mM.

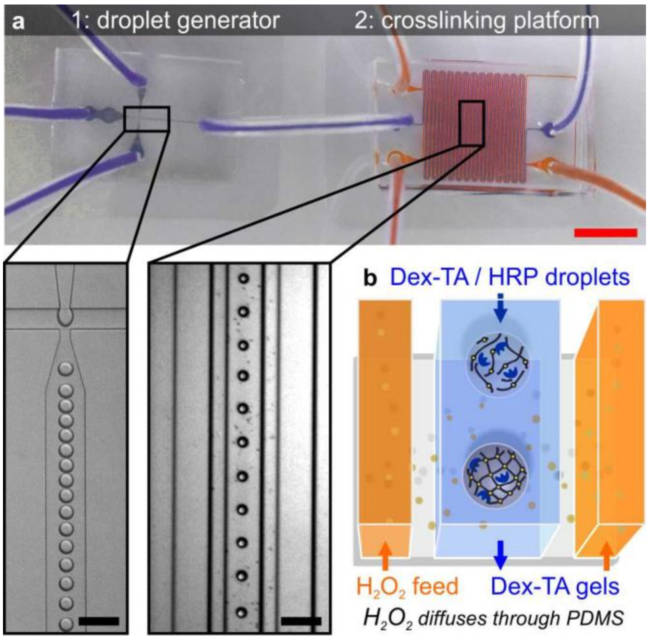

Figure S3. Droplet microfluidics-based microgel production. (**a**) To produce microgels, Dex-TA or Dex-TAB, and HRP containing hydrogel precursor solution was emulsified with surfactant containing oil using a standard microfluidic droplet generator that was connected to a microfluidic crosslinking platform, which consisted of three parallel channels separated by thin H_2O_2 permeable walls. (**b**) Crosslinking of hydrogel precursor microdroplets was induced with H_2O_2 diffusing from the outer channels through the polydimethylsiloxane (PDMS) walls and the oil phase into the center channel. This microgel production method has been previously described.[25] Scale bars indicate 5 mm (red) and 100 µm (black).

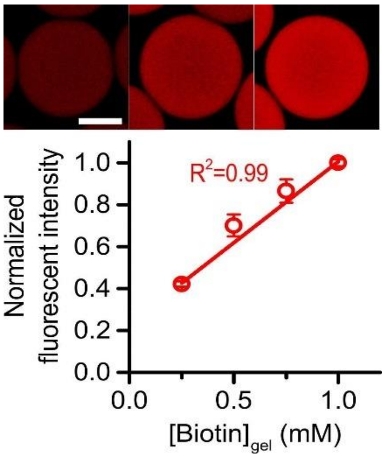

Figure S4. Tuning the biotin concentration in Dex-TAB microgels. The biotin concentration in Dex-TAB microgels could be tuned by mixing Dex-TA and Dex-TAB hydrogel precursor solutions, as previously described.[20] Subsequent coupling of multivalent neutravidin and fluorescently labeled biotin (biotin-atto565) revealed that the biotin concentration in the microgels linearly correlated (R^2 = 0.99) to the final degree of functionalization, as measured by the normalized fluorescent intensity. Fluorescent intensity data are given in means ± SD normalized to maximum average signal intensity (i.e., with 1 mM biotin), *n* = 8. Scale bar indicates 10 µm.

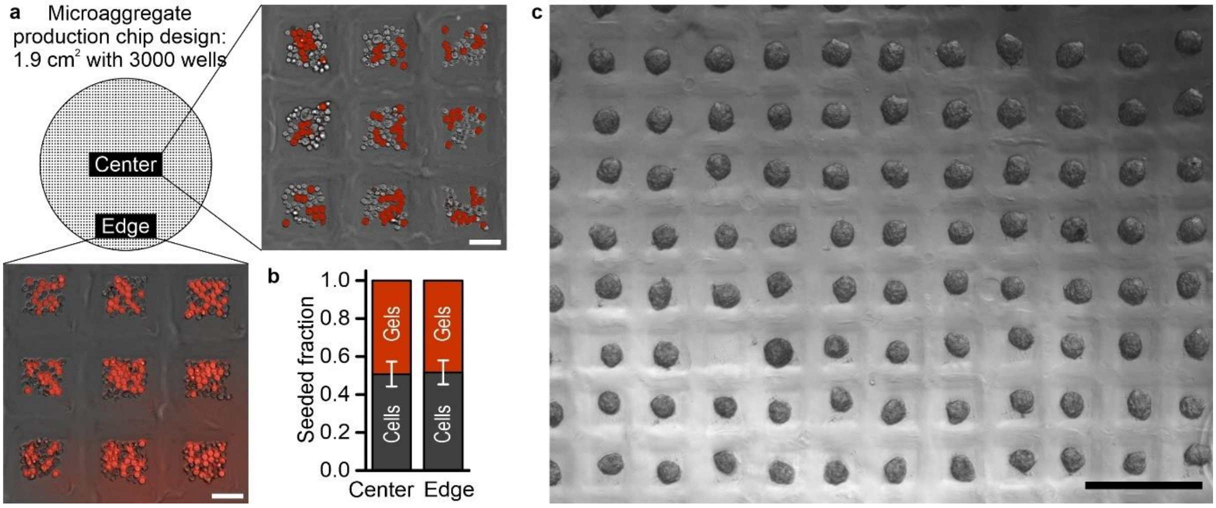

Figure S5. Homogeneous cell seeding in the microaggregate production chip. (**a**) To assess the seeding distribution of cells and microgels over the microwells, fluorescently labeled microgels (i.e., red) and cells were seeded in the microaggregate production chip. (**b**) Determining the per-well cell-to-gel ratios from microphotographs of the center and edge of the chip revealed that cells and gels were homogenously seeded in the microwells. Seeded fractions are given in means ± SD, n = 21. (**c**) Representative microphotograph after one day of culturing c(RGDfK)-functionalized microgels and MSCs. In each microwell, all cells and microgels self-assembled into composite microtissues with an average diameter of 110±10 µm. Scale bars indicate 100 µm (white) and 500 µm (black).

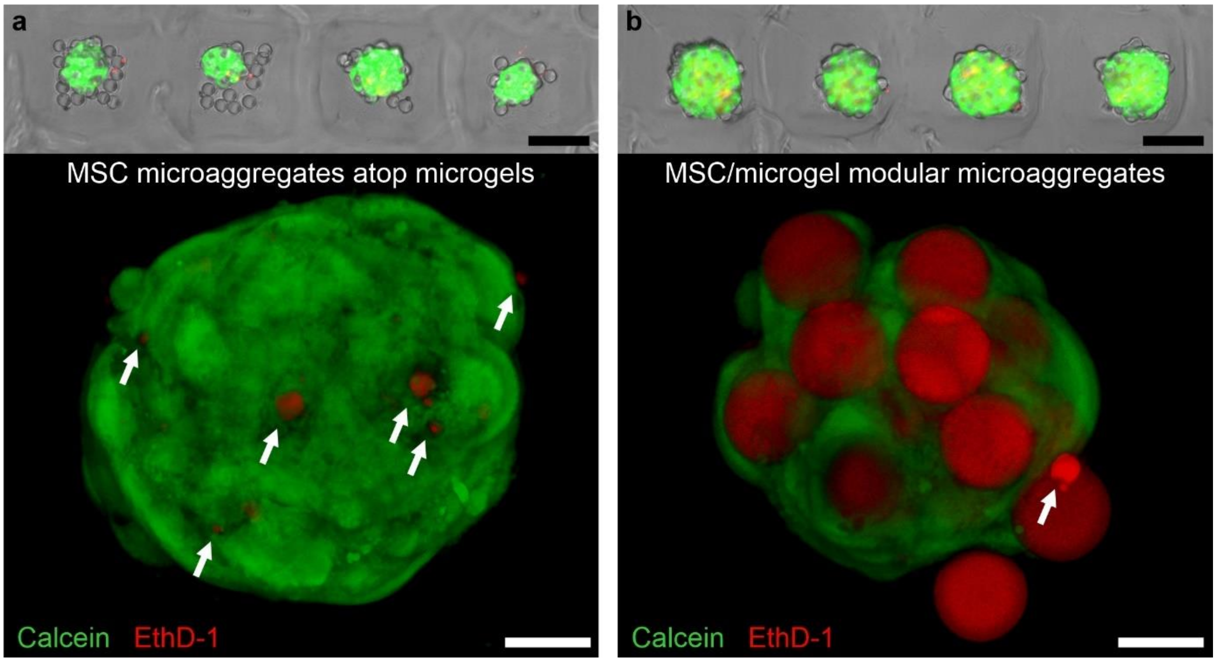

Figure S6. Composite microtissue viability. Microtissues consisting of (**a**) only MSCs and (**b**) MSCs and SCMs (50/50 ratio) were both characterized by > 90% cell viability, as demonstrated using live/dead staining comprising of Calcein AM (i.e., live cells in green) and homodimer-1 (EthD-1; i.e., dead cells in red). The top and bottom panels show representative live/dead stained microtissues as imaged using fluorescence microscopy and confocal fluorescence microscopy, respectively. Besides nuclei of dead cells, EthD-1 also stained Dex-TAB. Dead cells could be easily distinguished from the microgels based on staining intensity and size and are indicated with white arrows. Scale bars indicate 100 µm (black) and 20 µm (white).

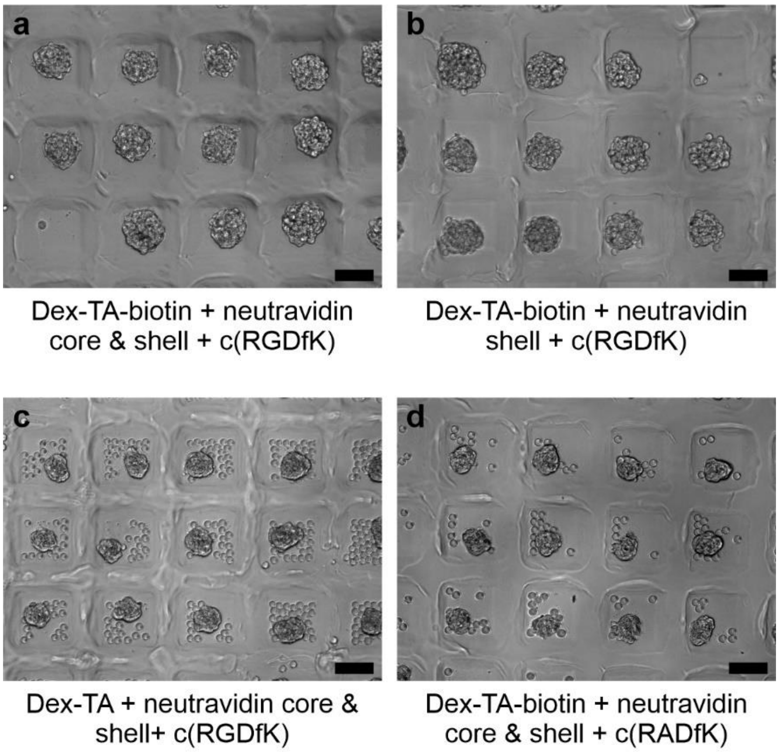

Figure S7. Tuning the microgels' cell-adhesive properties. (**a**) Dex-TAB microgels that were completely (i.e., core & shell) functionalized with c(RGDfK) peptides and (**b**) Dex-TAB microgels of which only the shell was functionalized with c(RGDfK) peptides readily adhered to cells, resulting in their complete incorporation into composite microtissues when seeded in a 50/50 ratio with MSCs. (**c**) Dex-TA (i.e., without biotin) microgels that were treated with the same neutravidin/c(RGDfK) functionalization protocol were not cell-adhesive and therefore did form self-assembled composite microtissues when seeded together with MSCs in a 50/50 ratio. (**d**) Also functionalization of Dex-TAB microgels with non-cell-adhesive c(RADfK) control peptides did not readily support cell adhesion nor subsequent incorporation of microgels into the composite microtissues, confirming that RGD-type peptides where essential to endow microgels with good cell-adhesive properties. Scale bars indicate 100 µm.

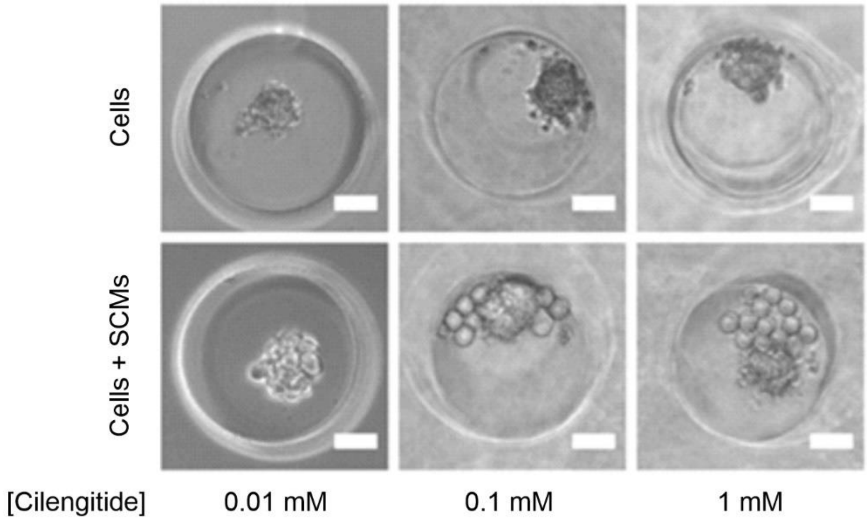

Figure S8. Effect of integrin blocker on composite microtissue formation. The formation of composite microtissues was prevented by blocking the RGD-type interactions using the soluble cyclic RGD peptide Cilengitide (i.e., integrins $\alpha_v\beta_3$ and $\alpha_v\beta_5$ inhibitor).[29] Scale bars indicate 50 µm.

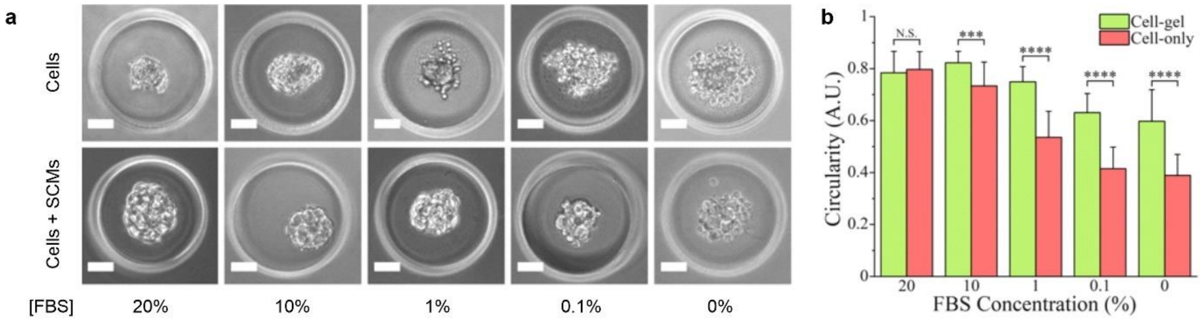

Figure S9. Effect of FBS concentration on composite microtissue formation. (**a,b**) Cell-only spheroids hardly formed stable spheroids with lower FBS concentrations, which was in sharp contrast to composite microtissues based on cells and SCMs containing 12 mM RGD (see **Figure S10**) that readily self-assembled, even in the absence of FBS. Circularity data are given in means ± SD, $n \geq 36$, significance is indicated (*** $p < 0.001$, **** $p < 0.0001$, 'n.s.' $p > 0.05$, one-way ANOVA with Tukey's post-hoc test). Scale bars indicate 50 µm.

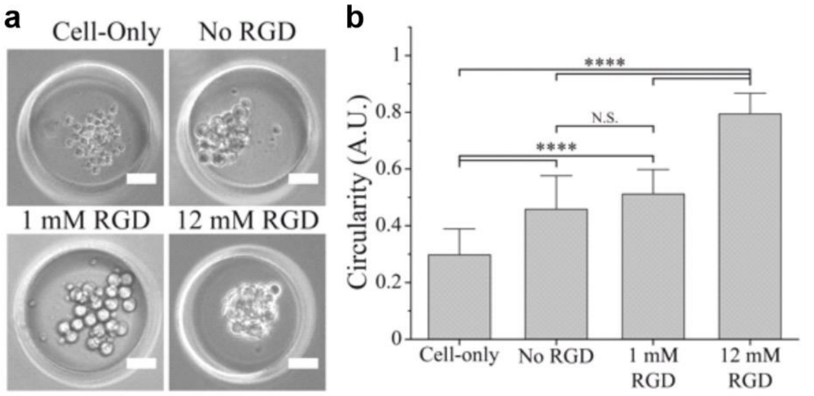

Figure S10. Effect of RGD concentration in SCMs on composite microtissue formation. (**a,b**) The self-assembly of cells and SCMs in serum-free media could be rescued by increasing the microgels' biotin (and thus biotinylated c(RGDfK)) concentration to 12 mM. Circularity data are given in means ± SD, $n \geq 35$, significance is indicated (**** $p < 0.0001$, 'n.s.' $p > 0.05$, Mann-Whitney). Scale bars indicate 50 µm.

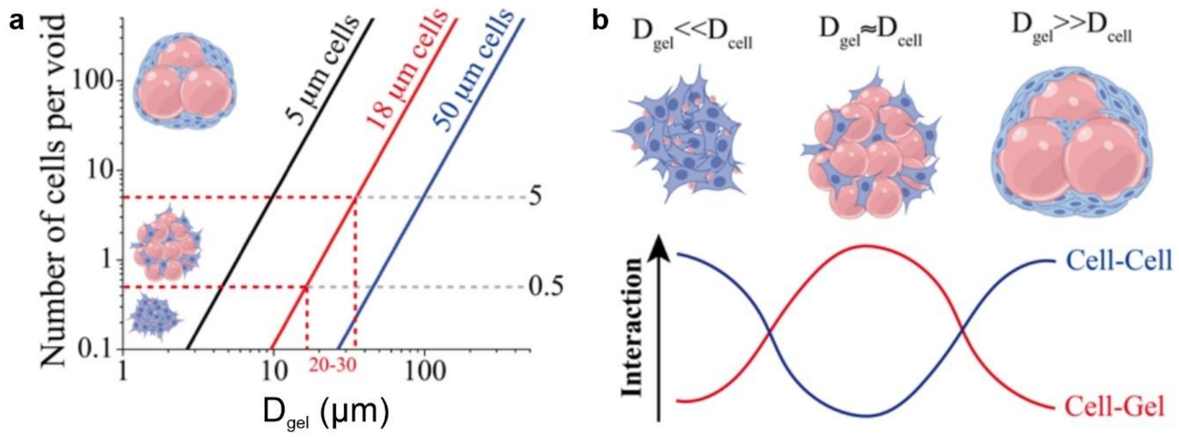

Figure S11. Tuning cell-cell and cell-material interactions via microgel size. (**a**) The number of cells per void as a function of microgel diameter were calculated assuming a random packing density of 60%. For example, randomly close packed microgels with a diameter between 20 µm and 30 µm have interstitial (i.e., inter-microgel) voids with a volume equaling 0.5 to 5 cells with a cell diameter of 18 µm. (**b**) Schematic depiction of the influence of microgel size on the balance between cell-cell and cell-material interactions.

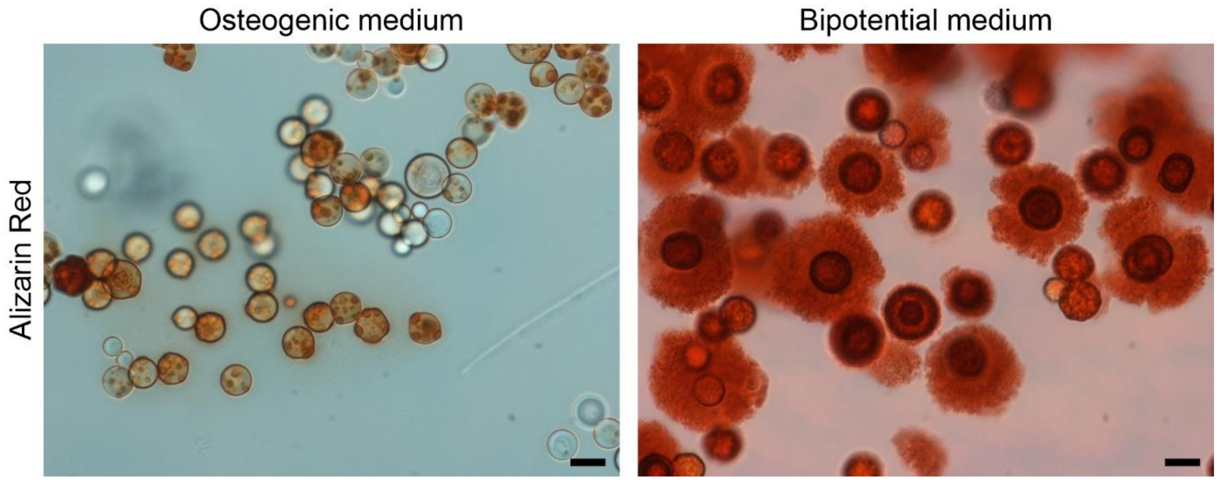

Figure S12. Comparison of osteogenic stem cell differentiation in Dex-TA microgels using osteogenic versus bipotential medium. MSC-laden microgels were cultured for three weeks in osteogenic or bipotential medium. Calcified matrix was stained with Alizarin Red. Scale bars indicate 50 µm.

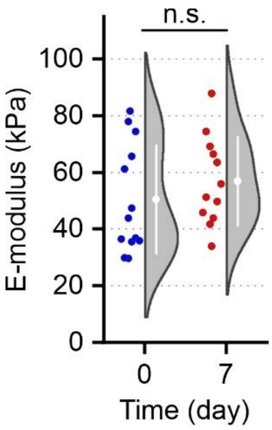

Figure S13. MSCs do not affect the Dex-TA microgel elasticity within seven days of culture. The E-modulus of Dex-TA microgels did not significantly change when cultured together with MSCs during seven days, as measured using nano-indentation. E-modulus data are presented as raw datapoints (left) and a Kernel density estimation using Scott's bandwidth smoothing with superimposed means ± SD (right), $n \geq 12$, significance is indicated ('n.s.' $p > 0.05$, Mann-Whitney).

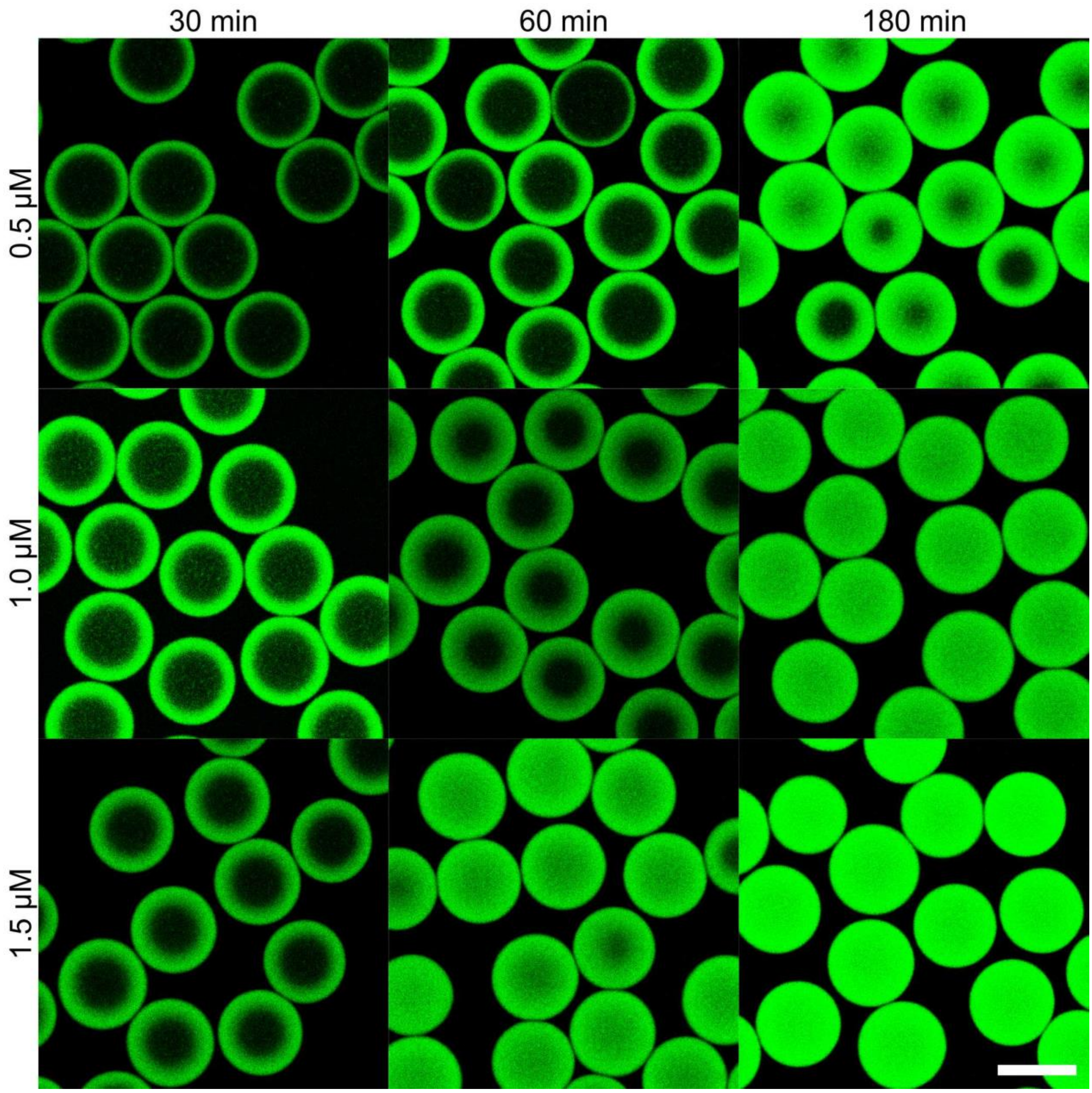

Figure S14. Controlling functionalized shell thickness of Dex-TAB microgels. Dex-TAB microgels could be endowed with a functional shell of tunable thickness by controlling the concentration and incubation time of neutravidin. The neutravidin acted as a template for subsequent tethering of biotinylated molecules of interest, as demonstrated using biotin-FITC (i.e., green). Scale bar indicates 20 µm.

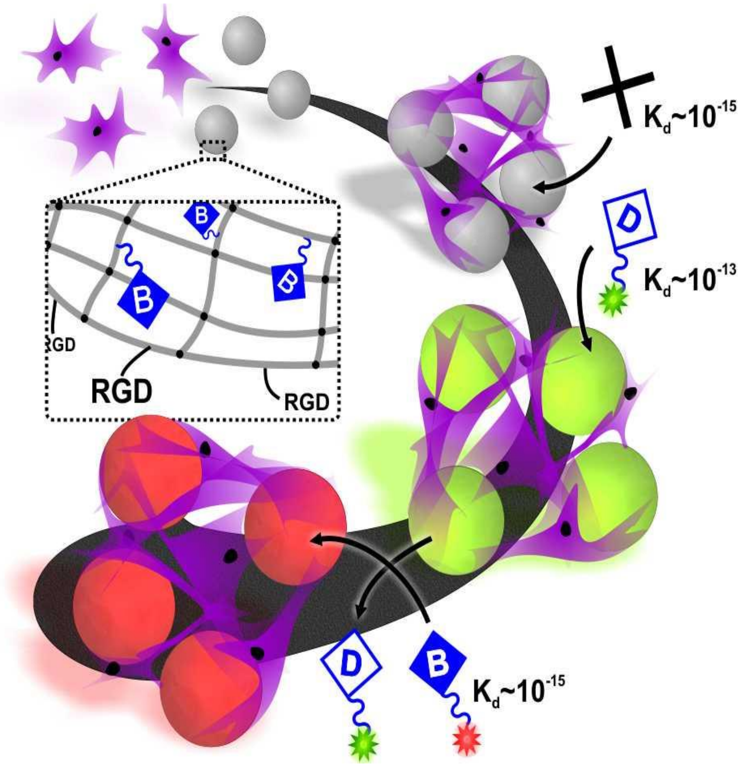

Figure S15. The concept of SCMs for engineering tunable 3D composite microtissues constructs. The shell of Dex-TAB microgels are permanently functionalized with cell-binding motives (RGD-type) to enable modular tissue engineering via the self-assembly of SCMs (grey) and cells (magenta). The composite microtissues can be further functionalized *in situ* via a two-step approach using tetravalent avidin (X) as the supramolecular linker between the SCMs' free biotins and desthiobiotinylated molecules of interest (green). Subsequently, the desthiobiotin can be displaced by biotinylated molecules of interest (red), thereby enabling on-demand biochemical tuning of the engineered composite microtissue.

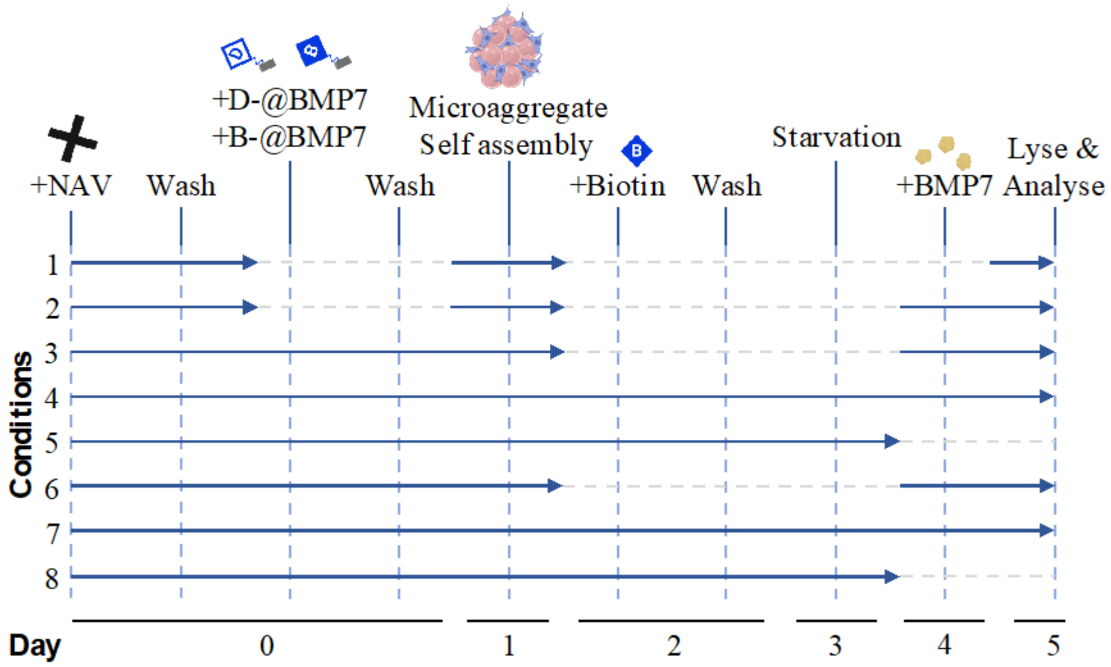

Figure S16. Stepwise experimental outline for the BMP7 induction experiment.
